# Biomarkers for diagnosis of stage III, grade C with molar incisor pattern periodontitis in children and young adults: a systematic review and meta-analysis

**DOI:** 10.1007/s00784-023-05169-x

**Published:** 2023-08-03

**Authors:** Meaad M. Alamri, Georgios N. Antonoglou, Gordon Proctor, Carlos Balsa-Castro, Inmaculada Tomás, Luigi Nibali

**Affiliations:** 1 Centre for Host Microbiome Interactions, Faculty of Dentistry, Oral and Craniofacial Sciences, King’s College London, London, UK; 2 Dental Health Department, College of Applied Medical Sciences, King Saud University, Riyadh, Kingdom of Saudi Arabia; 3 Centre for Dental Education, Faculty of Dentistry, Oral and Craniofacial Sciences, King’s College London, London, UK; 4 Oral Sciences Research Group, Department of Surgery and Medical‐Surgical Specialties, School of Medicine and Dentistry, Health Research Institute Foundation of Santiago (FIDIS), Universidade de Santiago de Compostela, Santiago de Compostela, Spain

**Keywords:** Stage III grade C, Juvenile, Aggressive, Periodontitis, Molecular biomarkers, Saliva, GCF, Peripheral blood, Serum, Interleukins, MMP

## Abstract

**Aim:**

To explore the existing salivary, gingival crevicular fluid (GCF), blood, and serum biomarkers associated with grade C molar-incisor pattern (C/MIP) periodontitis in systemically healthy children and young adults.

**Materials and methods:**

Cross-sectional, case–control, and cohort studies on stage III grade C periodontitis or former equivalent diagnosis with analysis of molecular biomarkers in saliva, GCF, blood, or serum were retrieved from six databases and screened based on the eligibility criteria. The risk of bias in included studies was evaluated. Meta-analysis was planned for biomarkers assessed using the same detection methods and sample type in at least two papers.

**Results:**

Out of 5621 studies identified at initial screening, 28 papers were included in the qualitative analysis of which 2 were eligible for meta-analysis for IgG in serum samples. Eighty-seven biomarkers were assessed with the majority being higher in cases than in controls. Only the meta-analysis of total serum IgG with low heterogeneity value revealed a significant increase in its levels in C/MIPs compared to controls (standardised mean difference: 1.08; 95% CI: 0.76, 1.40).

**Conclusion:**

There is a paucity of data on biomarkers associated with molar-incisor pattern periodontitis. Although serum IgG levels are raised, other more specific biomarkers in saliva, GCF, and blood/serum may be promising but require further investigation.

**Supplementary Information:**

The online version contains supplementary material available at 10.1007/s00784-023-05169-x.

## Introduction

Stage III grade C molar-incisor pattern (C/MIP) was formerly known as localised juvenile periodontitis (LJP), and then later as localised aggressive periodontitis (LAgP) [1, 2]. C/MIP is a chronic progressive inflammatory disease of the periodontium characterised by rapid destruction of the soft and hard tissue at an early age resulting in clinical attachment loss and bone resorption leading to tooth loss and functional impairments [3–6]. It affects the incisors and molars at first; thus, it was identified as a molar-incisor pattern (MIP) in the 2017 classification of periodontal diseases [3–7].

Unlike other periodontal diseases linked to plaque accumulation and poor oral hygiene over time, C/MIP is believed to have a strong genetic predisposition [8]. However, a better understanding of causative factors and specific pathogenic mechanisms still needs to be achieved. Systemically healthy and medically compromised children and young adults with familial aggregation can develop C/MIP at an early age [9, 10]. This condition increases the risk of premature tooth loss that negatively impacts individuals physically, psychologically, and aesthetically [11]. Therefore, early detection and treatment are of great importance [12].

Periodontal diagnosis is a crucial step in the oral examination as it affects the treatment plan and prognosis and influences the quality of life if not detected earlier [13]. Biomarkers in saliva, gingival crevicular fluid (GCF), peripheral blood, and serum might be used as indicators to diagnose periodontal diseases [13–15]. A previous systematic review/analysis study has confirmed the diagnostic accuracy of biomarkers in the detection of periodontitis, which may reflect their usefulness in the early detection or assessment of the risk of developing this pathology [16].

Saliva and GCF samples can be collected non-invasively and easily while GCF flow is collected and measured using sterile strips and a Periotron micro-moisture meter [15]. Saliva and GCF have different compositions and harbour host-derived markers [17]. In the presence of inflammation, saliva tends to have a higher concentration of defence factors such as immunoglobulin A (IgA), immunoglobulin G (IgG), and immunoglobulin M (IgM) [18], and the GCF flow increases as a host defence to eliminate the pathogens [19]. Moreover, some promising biomarkers of periodontitis were suggested, such as matrix metalloproteinase-8 (MMP-8), matrix metalloproteinase-9 (MMP-9), interleukin 1 beta (IL1β), and interleukin 6 (IL6) [2, 16, 20].

Peripheral blood and serum samples could also potentially be used as a source of biomarkers [21, 22]. Studies have shown a higher neutrophil–lymphocyte ratio (NLR), a lower lymphocyte-monocyte ratio (LMR) [14], increased levels of proinflammatory cytokines such as interleukin 17 (IL‐17) [21, 23], C-reactive protein (CRP), and fibrinogen in patients with periodontitis compared to healthy controls [24, 25]. Thus, these were considered potential biomarkers that need further affirmation [23].

However, to our knowledge, there are no studies that systematically evaluate biomarkers specifically associated with C/MIP. Discovering specific biomarkers for this condition might help in screening and identifying affected individuals at an early age, and it might help clarify pathogenic mechanisms. Therefore, the present systematic review aimed to explore the existing salivary, GCF, blood, and serum biomarkers used to diagnose C/MIP periodontitis in systemically healthy children and young adults.

## Materials and methods

The protocol was registered in the International Prospective Register of Systematic Reviews (PROSPERO) with ID no. CRD42022312530. This systematic review and meta-analysis were designed based on the Cochrane Handbook for Systematic Reviews of Diagnostic Test Accuracy and Preferred Reporting Items for Systematic Reviews and Meta-Analysis (PRISMA). The checklist can be found in the appendix (Appendix 1).

### PECOS question

The research question is “In subjects with stage III grade c with molar incisor pattern periodontitis, do the biomarker levels in body fluids differ compared to subjects with healthy periodontium?”.

The population (P), exposure (E), comparison (C), outcome (O), and study design (S) were as follows:

- P: systemically healthy children and young adults (≤ 25 years of age)
- E: stage III grade C with molar incisor pattern periodontitis or previous equivalent definitions
- C: healthy periodontium
- O: levels of salivary, GCF, peripheral blood, and serum biomarkers
- S: case–control studies, cross-sectional studies, cohort studies

### Eligibility criteria

Studies were included or excluded based on the following criteria.

#### Inclusion criteria

- Types of studies: cross-sectional, case–control, and cohort studies with analysis of molecular biomarkers
- Participants: a minimum of 10 systemically healthy children and young adults aged 25 years and younger in the case group
- Target condition: according to the 2017 classification of periodontal diseases, the target condition is stage III grade C periodontitis with molar incisor pattern or previous equivalent definitions, including early-onset periodontitis (EOP), aggressive periodontitis (AgP), juvenile periodontitis (JP), and rapidly progressive periodontitis (RPP) in both extents generalised and localised
- Case reference standard: stage III grade C is clinically defined as clinical attachment loss (CAL) ≥ 5, radiographic bone loss (RBL) extending to the middle third of root and beyond, ≤ 4 tooth loss due to periodontitis, in addition to probing depth (PD) ≥ 6 mm, vertical bone loss ≥ 3 mm, furcation involvement class II or III, and/or moderate ridge defects, progression of CAL or RBL of ≥ 2 mm over 5 years, percentage of bone loss by age is > 1 and tissue destruction exceeding the expectations given biofilm deposits [7]
- Control condition: healthy periodontium
- Control reference standard: no clinical evidence of periodontal disease
- Samples: saliva, GCF, blood, and/or serum
- Index test: molecular biomarkers identified in the samples of interest

#### Exclusion criteria

- Types of studies: cross-sectional, case–control, or cohort studies with genetic or microbiology profiles, randomised clinical trials (RCTs), case reports, reviews, non-clinical, in vitro, animal, and retracted/withdrawn studies were excluded
- Participants: subjects with systemic conditions, older than 25 years of age or with an unclear age range, recruited less than ten subjects in the case group, pregnant and lactating females, and smokers
- Definitions: non-C/MIP periodontitis
- Samples: swabs, gingival tissues, mouthwash, and plaque

### Search methods for identification of studies

#### Search strategy

The following databases were electronically searched from their oldest records until 08 February 2023: Embase (via Ovid), PubMed (MEDLINE), Web of Sciences (WoS), Scopus, and Virtual Health Library. Additionally, peer-reviewed digital dissertations (searched via UMI Proquest) were searched. The search was not restricted to papers in English, and no filters were applied.

The search strings were formulated to include the target condition, index test, type of samples, and population (Appendix 2).

### Data collection and analysis

#### Selection of studies

The papers retrieved from the six databases were de-duplicated following the Bramer et al. method [26]. Two reviewers (authors MA and GNA) independently screened the titles and abstracts of studies to identify articles that potentially meet the inclusion criteria. A pilot screening of 50 studies was done, and the results were compared to ensure consistency between reviewers. The full text of the potentially eligible studies and those abstracts that do not provide sufficient information to allow decision-making regarding inclusion or exclusion were retrieved, and the full texts were screened independently by GNA and MA. Any differences between the two reviewers were settled by consensus after consulting a third review author (LN).

#### Data extraction and management

*Relevant data from the included studies were independently* extracted by MA and GNA using a specifically designed extraction Excel form.

The following data were recorded for each study: study characteristics (author(s), year of publication, title, country, study design, setting, funding), demographics in cases and controls (number of periodontal disease cases and non-periodontal disease controls in the beginning and at the end, age, gender, ethnicity, and smoking status), definitions (periodontitis classification in the study, stage III grade C, and health periodontium), types of samples (saliva, GCF, peripheral blood, serum), biomarker detection methods, assessed biomarkers (name, class, biomarker levels—mean and standard deviation—and concentration units).

In longitudinal studies, only baseline data which is the first determination of the levels of biomarkers before treatment were collected and analysed. The mean and standard deviation were calculated if it was not reported. The mean was calculated by dividing the sum of values by the number of values and the standard deviation by multiplying the SE/SEM by the square root of N. In case 2SEM was given, SD was calculated by dividing 2SEM by 2 and then multiplying it by the square root of N.

#### Assessment of methodological quality

Two tools were used independently by MA and GNA to assess the risk of bias in the included studies: the Newcastle Ottawa Quality Assessment tool (NOS) for case–control and cohort studies and the modified version for cross-sectional studies. Results were compared for consistency, and variations were discussed and agreed on.

#### Statistical analysis and data synthesis

Meta-analysis was planned for biomarkers assessed using the same detection methods and sample types in at least two papers. The free software environment R (version 4.2.2) was used to analyse and create the meta-analysis (MA) models. Two types of models were run: models with a single standardised mean difference for each paper and models that included 2 or more standardised mean differences from the same paper [27, 28]. In the latter, the raw data from the same paper can be pooled, but to mitigate the risk of a unit-of-analysis error and to avoid “double counting” in the MA, it was necessary to pool the raw data with the dmetar package [29]. The meta package [30] was then used to obtain the random effects models and their *p*-value, the forest plots, and all statistics related to the between-study heterogeneity (*Q*-test, I2, H2, Tau2, Tau) of the 34 models obtained (18 non-pooled models and 16 pooled models). The restricted maximum likelihood (“REML”) [31] method was used in all models to calculate TAU2. The Hedges method was used in the MA models to estimate the standardised mean difference, thus avoiding overestimation bias due to the small number of studies included here.

## Results

**Fig. 1:**
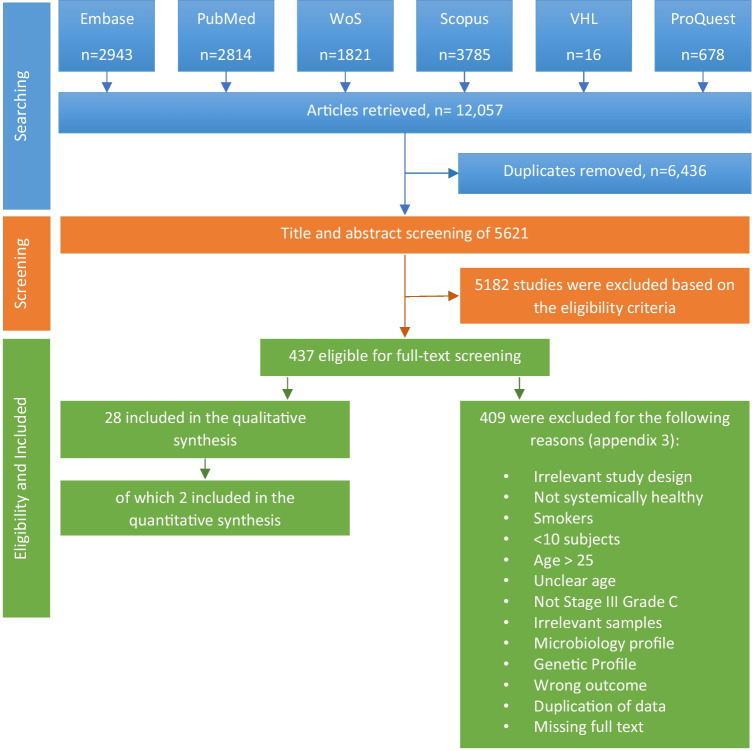
Flowchart of the search strategy

The total number of references retrieved after the removal of duplicates was 5621. Based on the title and abstract screening, 437 articles were eligible for full-text screening. Four hundred and nine articles were excluded for the reasons mentioned in the appendix 3, and 28 studies were included in the current review (Fig. 1). The kappa score and percentage of agreement for the abstract screening were respectively 0.766 and 98.7% and 0.813 and 97.2% for the full-text screening.

### Characteristics of included studies

The majority of studies had a case–control design (71.4%), while 14.3% were cohort and 14.3% were cross-sectional studies. Publication years ranged from 1974 to 2022, and most studies were conducted in the USA (*n* = 15), while others were conducted in Turkey (*n* = 4), Argentina (*n* = 2), and one study in each of the following countries: Brazil, Czech Republic, Finland, Germany, Norway, Sweden, UK, and India. The age of included patients ranged from 5 to 25 years old. The study sample size ranged from 10 to 79 in the cases and 5 to 103 in the controls. The definition of periodontal disease was based on clinical examination and/or radiographs to determine the presence/absence of CAL, PD, and RBL. Most of the definitions were based on the presence of bone loss (*n* = 16), CAL ≥ 2 and PD ≥ 5 (*n* = 5), CAL ≥ 3 (*n* = 3), PD ≥ 6 (*n* = 1), PD ≥ 4 (*n* = 1), PD ≥ 3 (*n* = 1), while one did not report a definition but it was included because they referred to C/MIP as “localized juvenile periodontitis” and used clinical indices: plaque index (PI), gingival index (GI), and probing depth (PD) to determine that condition of the periodontium. Definitions of periodontal health were based on not having evidence of bone loss, no bleeding on probing except one study stated that < 10% was accepted, and PD thresholds varied: PD < 4 (*n* = 2), PD ≤ 3 (*n* = 3), PD ≤ 2 (*n* = 3), and some did not specify a measurement (*n* = 20). Most studies focused on biomarkers in serum (*n* = 13) and GCF (*n* = 9), some in saliva (*n* = 5), and a few in blood (*n* = 3) and plasma (*n* = 3). Assays used included ELISA (*n* = 8), radial immunodiffusion (RID) (*n* = 5), Luminex multiplex immunoassay (*n* = 4), fluorometric immunoassay (*n* = 3), chromogenic immunoassay (*n* = 2), and the remaining studies used one of the following: checkerboard immunoblotting, electroimmunoassay, electroimmunodiffusion, indirect and direct immunofluorescence, luminol-dependent chemiluminescence immunoassay, lysis inhibition, and gamma spectroscopy, and radioimmunoassay, Table 1.

**Table 1:** Characteristics of the 28 studies included in the qualitative and quantitative analysis

No	Study ID*	Author, year of publication	Title	Country	Study design	N case/control	Age case control	Gender case M/F control M/F	Definitions of C/MIP control	Samples	Detection methods	Biomarkers	Meta-analysis
1	40	Branco-de-Almeida, 2020^**^	Treatment of Localized Aggressive Periodontitis Alters Local Host Immunoinflammatory Profiles: A Long-Term Evaluation	USA	Cohort	66/66	7–21 7–21	24/42 24/42	Aggressive: ≥ 2 sites (incisor and/or first molar, in permanent or primary dentition) with PD ≥ 5 mm, BOP, CAL ≥ 2 mm, and RBL including patients presenting with stages II and III, grade C periodontitis MIP Healthy: PD < 4 mm and no BoP, CAL or RBL	GCF	Luminex multiplex immunoassay	Eotaxin, GM-CSF IFN-γ, IL-1β, IL-2, IL-6, IL-8, IL-10 IL-12p40 IL-12p70 IP-10, MCP-1 MIP-1α, TNF-α RANKL, OPG	No
2	37	Albandar,1998	Crevicular Fluid Level of Beta-glucuronidase in Relation to Clinical Periodontal Parameters and Putative Periodontal Pathogens in Early-onset Periodontitis	USA	Cohort	64,24,58 /103	19–25 for all	NR	LEOP: having ≥ 4 teeth with CAL ≥ 3 mm or having ≥ 2 teeth with CAL ≥ 3 mm and had lost 3–11 incisors and molars. Primarily affecting the incisors and first molars and with only ≤ 2 of the affected teeth are cuspids, bicuspids or second molars GEOP: teeth with CAL similar to that of the LEOP group but > 2 of the affected teeth were cuspids, bicuspids or second molars Incidental EOP: individuals who did not meet the criteria of GEOP or LEOP and had ≥ 1 teeth with ≥ 3 mm attachment loss Healthy: no teeth having CAL ≥ 3 mm	GCF	Fluorometric immunoassay	Beta-glucuronidase	No
3	42	Goncxalves,2013^**^	Periodontal Treatment Reduces Matrix Metalloproteinase Levels in Localized Aggressive Periodontitis	USA	Cohort	29/29	5–21 5–21	11/18 11/18	Aggressive: ≥ 2 teeth (incisor or first molar and ≤ 2 other teeth other than first molars and incisors) presenting PD ≥ 5 mm with BOP, CAL ≥ 2 mm, and RBL Healthy: PD ≤ 3 mm with no BOP	GCF	Fluorometric immunoassay	MMP-1, MMP-2 MMP-3, MMP-8 MMP-9, MMP-12 MMP-13	No
4	47	Kalash,2015^***^	Influence of Periodontal Therapy on Systemic Lipopolysaccharides in Children With Localized Aggressive Periodontitis	USA	Cohort	25/NA	5–21 NA	9/16 NA	Aggressive: ≥ 2 teeth [incisor or first molar], PD ≥ 5 mm with BOP, CAL ≥ 2 mm, and RBL Healthy: NA	Plasma	Chromogenic assay	LPS	No
5	34	Akalin,1993	Beta 2-Microglobulin Levels in Serum and Saliva of Patients with Juvenile Periodontitis	Turkey	Case–control	11/10	14–23 22–27	1/10 3/7	Juvenile: deep PD and advanced vertical bone loss around incisors and first molars Healthy: NR	Saliva Serum	ELISA	Beta 2-Microglobulin	No
6	81	Celenligil, 1990	Juvenile and Rapidly Progressive Periodontitis. Peripheral Blood Lymphocyte Subpopulations	Turkey	Case–control	38/30	15–23 17–34	4/34 13/17	Juvenile: Baer 1971 Healthy: no clinical evidence of periodontal disease	Blood	Monoclonal antibodies and indirect and direct immunofluorescence	Lymphocytes B-cells CD3 + cells CD4 + cells CD8 + cells	No
7	51	Dibart, 1998	Rapid Evaluation of Serum and Gingival Crevicular Fluid Immunoglobulin G Subclass Antibody Levels in Patients with Early-Onset Periodontitis Using Checkerboard Immunoblotting	USA	Case–control	19/5	13–18 12–30	NR	EOP: American Academy of Periodontology (AAP) Healthy: NR	GCF^#^ Serum	Checkerboard immunoblotting	IgG_1_, IgG_2_ IgG_3_, IgG_4_	No
8	43	Monteiro,2020	The Familial Trend of the Local Inflammatory Response in Periodontal Disease	Brazil	Case–control	18/18	6–12 6–12	10/8 10/8	Aggressive: Armitage 1999 Healthy: no history of periodontal disease	GCF	Luminex multiplex immunoassay	IFN-γ, TNF-α IL-1β, IL-4 IL-6, IL-8 IL-10, IL-17	No
9	53	Schenck,1989	Serum Levels of Antibodies Against Actinobacillus Actinomycetemcomitans in Various Forms of Human Periodontitis	Norway	Case–control	10/9	13–20 > 20	NR	Juvenile: periodontal bone loss of at least one-third of the root length around ≥ 1 permanent first molar or incisor, as judged with a Schei ruler on intra-oral radiographs Healthy: no clinical and radiographic evidence of periodontal breakdown, and with < 10% BoP	Serum	ELISA	IgG to *Aa* IgG to *P. gingivalis* IgG to *B. Fragilis* IgA to *Aa* IgA to *P. gingivalis* IgA to *B. Fragilis* IgM to *Aa* IgM to *P. gingivalis* IgM to *B. Fragilis*	No
10	27	Shaddox,2011	Local Inflammatory Markers and Systemic Endotoxin in Aggressive Periodontitis	USA	Case–control	34/10	5–20 5–20	NR	Aggressive: ≥ 2 teeth presenting PD ≥ 5 mm, BoP, CAL ≥ 2 mm, and RBL Healthy: PD ≤ 3 mm, no BoP	GCF Plasma	Luminex multiplex immunoassay Chromogenic Assay	MCP-1, MIP-1α TNF-α, GM-CSF IFN-γ, IL-12p40 IL-1β, IL-2 IL-4, IL-6 IL-8, IL-10 LPS	No
11	32	Acquier,2017	Parameters of Oxidative Stress in Saliva From Patients with Aggressive and Chronic Periodontitis	Argentina	Case–control	20/20	17–23 17–23	10/10 10/10	Aggressive: Armitage 1999 Healthy: NR	Saliva	luminol-dependent chemiluminescence immunoassay	ROS TRAP TBARs	No
12	33	Acquier,2015	Comparison of Salivary Levels of Mucin and Amylase and Their Relation With Clinical Parameters Obtained From Patients With Aggressive and Chronic Periodontal Disease	Argentina	Case–control	20/20	17–23 17–23	10/10 10/10	Aggressive: Armitage 1999 Healthy: NR	Saliva	Chromogenic assay	Mucin Amylase Protein	No
13	52	Johnson,1980	Immunopathology of periodontal disease. I. Immunologic profiles in periodontitis and juvenile periodontitis	USA	Case–control	10/10	14–20 22–42	9/1 2/8	Juvenile: PD ≥ 6 only around central incisors and first molars, loss of alveolar bone mesial to first molars and central incisors, onset of disease during puberty or adolescence, gingiva of relatively healthy appearance, good hygiene, and rapid progression of disease Healthy: free gingival grafts, gingival crevice not greater than 3 mm, good oral hygiene, no clinical evidence of inflammation	Serum	Radial immunodiffusion (RID)	Serum IgG Serum IgA Serum IgM Serum C3 Serum C4	Yes
14	28	Albandar,2001	Associations Between Serum Antibody Levels to Periodontal Pathogens and Early-Onset Periodontitis	USA	Case–control	51, 13, 33 /62	19–25 for all	NR	GEOP. Individuals with ≥ 3 mm attachment loss affecting ≥ 4 teeth including ≥ 3 s molars, premolars, and cuspids; or who had lost ≥ 3 molars and incisors, and also had ≥ 2 teeth with ≥ 3 mm CAL or ≥ 1 teeth with ≥ 4 mm CAL of which ≥ 3 teeth were second molars, premolars, and cuspids LEOP: ≥ 3 mm CAL in ≥ 4 teeth including ≤ 2 s molars, premolars, and cuspids; or who had lost 3 to 11 molars and incisors and, in addition, had ≥ 2 teeth with ≥ 3 mm CAL, or had ≥ 1 teeth with ≥ 4 mm CAL of which ≤ 2 teeth were second molars, premolars, and cuspids Incidental EOP: individuals not meeting the criteria of LOEP or GEOP and who had 1 or more teeth with ≥ 3 mm attachment loss were classified in the incidental EOP group Healthy: no teeth showing a CEJ to bottom of sulcus distance exceeding 2 mm	Serum	ELISA	IgG to *Aa* IgG to *P. gingivalis* IgG to *P. intermedia* IgG to *C. rectus* IgG to *E. corrodens* IgG to *F. nucleatum* IgA to *Aa* IgA to *P. gingivalis* IgA to *P. intermedia* IgA to *C. rectus* IgA to *E. corrodens* IgA to *F. nucleatum* IgM to *Aa* IgM to *P. gingivalis* IgM to *P. intermedia* IgM to *C. rectus* IgM to *E. corrodens* IgM to *F. nucleatum*	No
15	49	Albandar,2002	Associations of Serum Concentrations of IgG, IgA, IgM and Interleukin-1beta With Early-Onset Periodontitis Classification and Race	USA	Case–control	51,13, 33/62	19–25 for all	NR	GEOP. Individuals with ≥ 3 mm attachment loss affecting ≥ 4 teeth including ≥ 3 s molars, premolars, and cuspids; or who had lost ≥ 3 molars and incisors, and also had ≥ 2 teeth with ≥ 3 mm CAL or ≥ 1 teeth with ≥ 4 mm CAL of which ≥ 3 teeth were second molars, premolars, and cuspids LEOP: ≥ 3 mm CAL in ≥ 4 teeth including ≤ 2 s molars, premolars, and cuspids; or who had lost 3 to 11 molars and incisors and, in addition, had ≥ 2 teeth with ≥ 3 mm CAL, or had ≥ 1 teeth with ≥ 4 mm CAL of which ≤ 2 teeth were second molars, premolars, and cuspids Incidental EOP: individuals not meeting the criteria of LEOP or GEOP and had one or more teeth with ≥ 3 mm attachment loss were classified in the incidental EOP group Healthy: no teeth showing a CEJ to bottom of sulcus distance exceeding 2 mm	Serum	Radioimmunoassay Radial immunodiffusion (RID)	IL-1β IgG, IgA, IgM IgG1, IgG2 IgG3, IgG4	No
16	38	Alfant,2008	Matrix Metalloproteinase Levels in Children With Aggressive Periodontitis	USA	Case–control	23/12	7–19 7–19	NR	Aggressive: AAP Deep sites: PD ≥ 4 mm Shallow sites: PD ≥ 2 mm Healthy: PD ≤ 2 mm	GCF	Fluorometric immunoassay	MMP-1, MMP-2 MMP-3, MMP-8 MMP-9, MMP-12 MMP-13	No
17	50	Anil,1990	Circulating Immune Complexes in Localised Juvenile Periodontitis	India	Case–control	15/15	13–21 13–21	6/9 6/9	Juvenile: based on Manson et al. 1974 and Zambon et al. 1976 established criteria: Radiographic advanced vertical bone destruction involving > 1 tooth most often affecting the permanent first molars and incisors, local etiological factors were not commensurate with the severity of the bone loss, and the patients were healthy and there was no relevant present or past general disease Healthy: PI < 0.2	Serum	Radial immunodiffusion (RID)	CIC IgG in CIC IgM in CIC	Yes
18	44	Celenligir,1998	Analysis of Serum Antibody Responses to Periodontopathogens in Early-onset Periodontitis Patients From Different Geographical Locations	USA and Turkey	Case–control	22/12	15–23 20–30	4/18 8/4	EOP: Ebersole1987 and celengili1990 criteria Healthy: no clinical evidence of periodontal disease	Serum	ELISA	IgG to *Aa* IgG to *P. gingivalis* IgG to *P. intermedia* IgG to *C. rectus* IgG to *E. corrodens* IgG to *F. nucleatum* IgG to *C.ochracea*	No
19	35	Fine,2013	Can Salivary Activity Predict Periodontal Breakdown in a. Actinomycetemcomitans Infected Adolescents?	USA	Case–control	10/20	12–17 12–17	NR	Aggressive: developed bone loss Healthy: NR	Saliva	ELISA	Lactoferrin Iron	No
20	41	Friedman,1983	Lysozyme and Lactoferrin Quantitation in the Crevicular Fluid	USA	Case–control	11/7	12–22 20–25	NR	Juvenile: Baer criteria which is RBL around 1st molars and anterior teeth Healthy: no radiographic evidence of bone loss	GCF	Electroimmunodiffusion (Rocket)	Lysozyme Lactoferrin	No
21	36	Lehner,1974	Immunological Aspects of Juvenile Periodontitis (Periodontosis)	UK	Case–control	23/26	14–21 NR	6/17 NR	Juvenile: Baer 1971 Localized disease, affecting otherwise healthy adolescents and young adults, and is characterized by a rapid loss of alveolar bone, about > 1 permanent tooth that cannot be accounted for by local factors Healthy: NR	Saliva Serum	Radial immunodiffusion (RID)	Saliva IgA Serum IgG Serum IgA Serum IgM	No
22	46	Sjödin,1995	Periodontal and Systemic Findings in Children With Marginal Bone Loss in the Primary Dentition	Sweden	Case–control	24/7	7–9 7–9	NR	Case: bone loss at ≥ 2 proximal tooth surfaces in the posterior areas of the primary dentition Healthy: clinically healthy appearance and without BOP	Serum Blood	Lysis inhibition assay and gamma spectroscopy	Serum IgG Serum IgA Serum IgM Serum ALP HB MCV LPK ANC TPK	No
23	55	Unsal,1996	Serum Antibodies to Actinobacillus Actinomycetemcomitans and Porphyromonas Gingivalis in Juvenile Periodontitis and Adult Periodontitis (Part I)	Turkey	Case–control	17/24	17–24 20–47	2/15 12/12	Juvenile: Baer 1971 Healthy: no evidence of RBL or gingivitis	Serum	ELISA	IgG to *Aa* IgG to *P. gingivalis* IgM to *Aa* IgM to *P. gingivalis*	No
24	48	Zafiropoulos,1987	Determination of the Elp (Elastase-like Proteinase) Plasma Levels in Patients With Rapidly Advancing and With Juvenile Periodontitis	Germany	Case–control	11/22	17–21 17–21	NR	Juvenile: NR Healthy: free of any oral or general disease	Plasma	ELISA	ELP-a-PI Complex ELP content	No
25	39	Bartova,1995^***^	Local Antibodies and Cytokine Responses in Crevicular Fluid of Patients With Juvenile Periodontitis	Czech Republic	Cross-sectional	20/NA	17–25 NA	NA	Juvenile: the presence of gingival inflammation, PD deeper than 3 mm, and *Aa* in the periodontal pockets Healthy: NA	GCF	ELISA	IgG IgA Ig to *Aa*	No
26	45	Sandholm,1983^***^	Concentrations of Serum Protease Inhibitors and Immunoglobulins in Juvenile Periodontitis	Finland	Cross-sectional	15/NA	15–24 NA	5/10 NA	Juvenile: Baer 1971 Healthy: NA	Serum Blood	Radial immunodiffusion (RID)	Serum α_2_M Serum IgG Serum IgA Serum IgM Serum total protein Differ. WBCC	No
27	54	Spindler,1985^***^	Juvenile Periodontitis I. Demonstration of Local Immunoglobulin Synthesis	USA	Cross-sectional	19/NA	13–21 NA	1/18 NA	Juvenile: Baer 1971 Healthy: NA	Serum	Electroimmunoassay	IgG/albumin ratio	No
28	82	Tavakoli, 2022	Gender differences in immunological response of African American juveniles with grade C molar incisor pattern periodontitis	USA	Cross-sectional	79/96	6–23 5–23	26/53 38/58	C/MIP: ≥ 2 teeth presenting PD ≥ 5 mm with BoP, CAL ≥ 2 mm, and RBL Healthy: absence of PD ≥ 5 mm with BoP, CAL ≥ 2 mm, and RBL	GCF Blood^##^	Luminex multiplex immunoassay	Eotaxin, IP10 IL-1β, IL-2 IL-6, IL-8 IL10, IL12(p40) G-CSF, GM-CSF IFNγ, MCP1 MIP1α, TNFα	No

^*^Study ID in the manuscript’s list of references, **studies assessed healthy and diseased sites from the same patients (no control group),***no control group in the study, **#**samples were excluded from analysis because it was collected from one patient, **##**blood samples were excluded from analysis because it was stimulated in the laboratory, *PD*, probing depth; *BoP*, bleeding on probing; *CAL*, clinical attachment loss; *RBL*, radiographic bone loss; *MIP*, molar incisor pattern; *GCF*, gingival crevicular fluid; *GM-CSF*, granulocyte–macrophage colony-stimulating factor; *IFN-γ*, interferon-gamma; *Interleukins*, IL-1β, IL-2, IL-6, IL-8, IL-10, IL-17 IL-12p40, IL-12p70; *IP-10*, interferon γ-induced protein 10 kDa; *MCP-1*, monocyte chemoattractant protein-1; *MIP-1α*, macrophage inflammatory protein-1 alfa; *TNF-α*, tumour necrosis factor-alfa; *RANKL*, receptor activator of NF-kappaB ligand; *OPG*, osteoprotegerin; *NR*, not reported; *LEOP*, localized early-onset periodontitis; *GEOP*, generalized early-onset periodontitis; *EOP*, early-onset periodontitis; *Matrix Metalloproteinase*, MMP-1, MMP-2, MMP-3, MMP-8, MMP-9, MMP-12, MMP-13; *NA*, not applicable; *LPS*, lipopolysaccharides; *ELISA*, enzyme-linked immunosorbent assay; *Immunoglobulins*, IgG1, IgG2, IgG3, IgG4; *IgG*, immunoglobulin G; *IgA*, immunoglobulin A; *IgM*, immunoglobulin M; *Aa*, *Aggregatibacter actinomycetemcomitans*; *P. gingivalis*, *Porphyromonas gingivalis*; *B. fragilis*, *Bacteroides fragilis*; *ROS*, reactive oxygen species; *TRAP*, total radical-trapping antioxidant potential; *TBARs*, thiobarbituric acid-reactive substances; *P. intermedia*, *Prevotella intermedia*; *C. rectus*, *Campylobacter rectus*; *E. corrodens*, *Eikenella corrodens*; *F. nucleatum*, *Fusobacterium nucleatum*; *CIC*, circulating immune complexes; *C. ochracea*, *Capnocytophaga ochracea*; *ALP*, alkaline phosphatase; *HB*, hemoglobin; *MCV*, mean corpuscular volume; *LPK*, leukocytes; *ANC*, absolute neutrophil count; *TPK*, tyrosine protein kinase; *ELP-α-PI complex*, ELP alfa proteinase inhibitor complex; *ELP*, elastase-like protease; *Serum α2M*, serum alpha-2-macroglobulin; *Differ. WBCC*, differential white blood cells; *C/MIP*, grade C molar incisor pattern

### Data extraction

In the screening phase, some abstracts had missing full text, so the authors were contacted to request the articles and review them against eligibility criteria. Similarly, when extracting the means and standard deviations for each biomarker, some papers presented their results in graphs/plots/charts without giving numerical values, so the dataset was requested from the authors. Not all authors were able to provide the requested datasets. None of the papers reported the sensitivity and specificity of the assessed biomarkers, and only one study gave the contingency tables.

### Biomarkers analysed

When grouping the studies by analysed biomarkers, some focused on cytokines such as interleukins IL-1β, IL-2, IL-4, IL-6, IL-8, IL-10, IL-12p40, IL-12p70, and IL-17, interferons: interferon-gamma (IFN-γ) and interferon γ-induced protein 10 kDa (IP-10), chemokines: monocyte chemoattractant protein-1 (MCP-1), eotaxin, macrophage inflammatory protein-1 alfa (MIP-1α), tumour necrosis factor-alfa (TNF-α), granulocyte colony-stimulating factor (G-CSF), and granulocyte–macrophage colony-stimulating factor (GM-CSF), others on tissue degradation markers such as matrix metalloproteinase MMP-1, MMP-2, MMP-3, MMP-8, MMP-9, MMP-12, and MMP-13, while others on serum immunoglobulins such as IgG, IgA, and IgM and immunoglobulins to specific pathogens. Additionally, blood cells, tumour markers, enzymes, proteins, antibodies, and some other molecules were assessed as listed in Table 1.

### Characteristics of studies in saliva

The following 10 salivary biomarkers were assessed in 5 case–control studies [32–36] from Turkey, the USA, Argentina, and the UK: β2-microglobulin, lactoferrin, iron, reactive oxygen species (ROS), total radical-trapping antioxidant potential (TRAP), thiobarbituric acid-reactive substances (TBARs), mucin, amylase, protein, and IgA. They were all higher in cases than in controls including the biomarkers without numerical data presented except for lactoferrin and iron. They were assessed in two groups of patients, one with *Aggregatibacter actinomycetemcomitans* and one without, and were followed over time to observe the development of the disease. Both lactoferrin and iron were lower in Aa-positive LAP subjects than in Aa-negative and positive healthy subjects. A meta-analysis was not possible due to the different salivary biomarkers used in the different studies.

### Characteristics of studies in GCF

Nine studies (three cohorts, four case-controls, and two cross-sectional) [27, 37–43] from the USA, Brazil, and Czech Republic investigated 31 biomarkers: eotaxin, IL-1β, IL-2, IL-4, IL-6, IL-8, IL-10, IL-12P40, IL-12p70, IL-17, IP-10, IFN-γ, MCP1, MIP-α, TNF-α, G-CSF, GM-CSF, receptor activator of NF-kappaB ligand (RANKL), osteoprotegerin (OPG), β-glucuronidase, MMP-1, MMP-2, MMP-3, MMP-8, MMP-9, MMP-12, MMP-13, lysozyme, lactoferrin, IgG, IgA, Ig to Aa. The majority of biomarkers were higher in cases than in controls except for MCP-1, which was higher in controls in 2 studies. Additionally, a few biomarkers such as IFN-γ, IL-4, IL-6, IL-8, IL10, IL-17, and TNF-α were higher in controls in some studies and lower in others (Table 2). Twelve meta-analyses were performed involving the following molecules: GM-CSF, IFN-γ, IL1-β, IL-2, IL-4, IL-6, IL-8, IL-10, IL-12p40, MCP-1, MIP-1α, and TNF-α. However, because of high heterogeneity (*I*^2^ > 75%), the meta-analyses are considered inconclusive (Appendix 4).

**Table 2:** Comparison of biomarkers between cases and controls in 28 studies

Author, year of publication study ID	Method	Biomarkers	Cases (μ, SD)		Control (μ, SD)		Unit
Saliva							
Akalin, 1993 34	ELISA	β2-microglobulin	2.08	0.46	1.29	0.28	mg/ml
Fine, 2013 35	ELISA	Lactoferrin*	Aa + , prior to disease: 268	81	Aa-H:2121 Aa + H:862	187 776	μg/dl
			Aa + After: > prior				
		Iron *	Before BL: 3.6 After BL: 4.1	1.7 3.1	Aa-H:65 Aa + H:150	NR	ng Fe/μg
Acquier, 2017 32	Luminol-dependent chemiluminescence immunoassay	ROS	7032	400	1082	64	RLU
		TRAP	(No numerical data presented)				NA
	Fluorometer	TBARs	(No numerical data presented)				
Acquier, 2015 33	Chromogenic assay	Mucin	(No numerical data presented)				NA
		Amylase					
		Protein					
Lehner, 1974 36**	Radial immunodiffusion (RID)	Saliva IgA	Caucasian: 9.52 Afro-Asian: 7.66	5.53 5.77	C:7.72 A:5.5	3.84 3.44	mg/ml
GCF							
Branco-de-Almeida, 2020 40	Luminex multiplex immunoassay	IL-1β	19.16	34.47	9.63	19.86	pg/mL
		IL-2	2.94	6.90	2.10	4.55	
		IL-6	1.56	1.54	1.25	1.10	
		IL-8	353.07	596.81	222.40	283.65	
		IL-10	2.44	3.67	1.62	1.55	
		GM-CSF	1.38	1.47	1.21	1.03	
		MCP-1*	7.61	7.77	10.79	17.85	
		MIP-1α	13.76	11.64	11.71	12.38	
		TNF-α*	1.85	3.51	1.91	2.26	
		IFN-γ	1.25	1.68	1.01	0.97	
		IL-12p40	7.34	7.99	5.41	6.44	
		IL-12p70	(No numerical data presented)				NA
		Eotaxin					
		IP-10					
		RANKL					
		OPG					
Monteiro, 2020 43	Luminex multiplex immunoassay	IL-1β	14.7	12.6	13.8	8.5	pg/mL
		IL-4*	11.5	5.7	13	11.8	
		IL-6*	8.6	4.0	18.02	17.0	
		IL-8	989.2	658.9	808.3	558.8	
		IL-10*	12.9	6.4	24.1	21.2	
		IL-17*	5.6	3.0	10.3	9.5	
		TNF-α	15.1	12.5	13.9	12.6	
		IFN-γ*	8.7	4.9	16.2	12.8	
Shaddox, 2011 27	Luminex multiplex immunoassay	MCP-1*	0.6542	0.5375	10.81	11.45	pg/mL
		MIP-1α	459.4	149.3	58.75	39.61	
		TNF-α	9931	1822	80.24	45.33	
		GM-CSF	840.3	507.9	75.79	68.28	
		IFN-γ	314	66.34	121.1	39.05	
		IL-1β	5429	2710	9.52	8.288	
		IL-2	2051	687.4	3.554	2.375	
		IL-4*	0.493	0.4988	1.303	0.8342	
		IL-6	13.12	8.228	7.476	5.182	
		IL-8*	181.7	143.6	305.7	233.4	
		IL-10	1423	181.7	20.5	20.42	
		IL-12p40	1104	272.7	103.9	45.97	
Tavakoli, 2022 82	Luminex multiplex immunoassay	IL-1β	(No numerical data presented)				NA
		IL-2					
		IL-6					
		IL-8					
		IL-10					
		IL-12p40					
		IP-10					
		G-CSF					
		GM-CSF					
		MCP-1					
		MIP-1α					
		TNF-α					
		IFN-γ					
		Eotaxin					
Albandar, 1998 37**	Fluorometric immunoassay	β-glucuronidase	G: 65.4 L: 50.8 I: 37	36.5 36.2 36.5	26	36.4	βG units
Alfant, 2008 38	Fluorometric immunoassay	MMP-1	(No numerical data presented)				NA
		MMP-2					
		MMP-3					
		MMP-8					
		MMP-9					
		MMP-12					
		MMP-13					
Goncxalves, 2013 42	Fluorometric immunoassay	MMP-1	(No numerical data presented)				NA
		MMP-2					
		MMP-3					
		MMP-8					
		MMP-9					
		MMP-12					
		MMP-13					
Friedman, 1983 41	Electroimmunodiffusion (Rocket)	Lysozyme	0.16	0.08	0.06	0.03	μg/lambda
		Lactoferrin	1.7	0.53	0.63	0.32	
Bartova, 1995 39	ELISA	IgG	(No numerical data presented)				NA
		IgA					
		Ig to *Aa*					
Whole blood							
Celenligil, 1990 81**	Monoclonal antibodies and indirect and direct immunofluorescence	Lymphocytes*	1817	927.7	2331	427.2	Absolute cell counts mm^−3^
		B-cell*	416	117.1	490	117.7	
		CD3 + cells*	956	228.08	1025	246.4	
		CD4 + cells*	552	126.3	733	172.5	
		CD8 + cells*	414	129.4	565	123.2	
Sandholm, 1983 45	Radial immunodiffusion (RID)	Differ. WBCC	8 cases had ↑lymph and 3 normal			NA	NA
Sjödin, 1995 46	Lysis inhibition assay and gamma spectroscopy	HB*	126	10	128	9	g/l
		MCV	83	4	81	3	fl
		LPK	6.9	1.6	5.5	1	10^9^/l
		ANC	3.7	1.3	2.5	0.6	10^9^/l
		TPK	288	81	252	52	10^9^/l
Plasma							
Shaddox, 2011 27	Chromogenic assay	LPS	(No numerical data presented)				NA
Kalash, 2015 47	Chromogenic assay	LPS	0.44	0.28	NA	NA	EU/ml
Zafiropoulos, 1987 48	ELISA	ELP-a-PI complex	(No numerical data presented)				NA
		ELP content					
Serum							
Akalin, 1993 34	ELISA	Beta 2-microglobulin	2.86	0.13	2.62	0.05	mg/ml
Schenck, 1989 53	ELISA	IgG to *Aa*	0.49	2.8	0.26	0.47	OD
		IgG to *P. gingivalis*	0.22	0.91	0.15	0.39	
		IgG to *B. Fragilis*	0.24	0.39	0.22	0.4	
		IgA to *Aa*	0.32	2.1	0.32	0.5	
		IgA to *P. gingivalis*	0.3	0.89	0.2	0.41	
		IgA to *B. Fragilis*	0.2	0.35	0.19	0.34	
		IgM to *Aa*	0.49	1.04	0.31	0.71	
		IgM to *P. gingivalis*	0.46	1.27	0.39	0.82	
		IgM to *B. Fragilis*	0.48	1.16	0.43	0.94	
Celenligir, 1998 44	ELISA	IgG to *Aa*	(No numerical data presented)				NA
		IgG to *P. gingivalis*					
		IgG to *P. intermedia*					
		IgG to *C. rectus*					
		IgG to *E. corrodens*					
		IgG to *F. nucleatum*					
		IgG to *C. ochracea*					
Unsal, 1996 55	ELISA	IgG to *Aa*	1.11	0.07	0.32	0.01	OD
		IgG to *P. gingivalis*	0.83	0.05	0.37	0.01	
		IgM to *Aa*	0.82	0.04	0.31	0.01	
		IgM to *P. gingivalis*	0.64	0.04	0.32	0.01	
Albandar, 2001 28	ELISA ELISA	IgG to Aa*	2.77 2.11 2.47	1.3 0.64 1.03	2.27	0.84	EU EU EU
		IgG to *P. gingivalis*	3.45 2.92 2.92	1.04 1.05 0.9	2.53	0.88	
		IgG to P. intermedia*	4.64 4.41 4.69	0.59 0.48 0.52	4.51	0.47	
		IgG to C. rectus*	4.58 4.54 4.42	0.76 0.54 0.63	4.46	0.93	
		IgG to E. corrodens*	4.41 4.05 4.38	0.69 0.44 0.48	4.39	0.51	
		IgG to F. nucleatum*	2.53 2.34 2.62	0.81 0.58 0.69	2.75	0.68	
		IgA to *Aa*	4.01 3.54 3.43	1.12 0.77 0.80	3.38	0.76	
		IgA to *P. gingivalis*	1.73 1.59 1.50	0.85 0.91 1.05	1.23	0.94	
		IgA to *P. intermedia*	4.31 4.00 4.03	0.90 0.89 1.09	3.72	0.90	
		IgA to C. rectus*	3.66 3.98 3.76	0.68 0.91 1.02	3.84	0.88	
		IgA to E. corrodens*	3.38 3.55 3.15	0.73 0.79 0.67	3.44	0.72	
		IgA to F. nucleatum*	3.48 3.71 3.71	0.77 0.84 0.79	3.66	0.85	
		IgM to* Aa*	4.67 4.92 4.78	0.86 0.82 0.84	4.37	0.86	
		IgM to P. gingivalis*	4.68 4.77 4.46	0.94 0.99 0.99	4.48	0.58	
		IgM to *P. intermedia*	4.85 5.31 4.88	0.84 0.47 0.89	4.8	0.93	
		IgM to *C. rectus*	5.03 5.26 5.12	1.02 0.65 0.95	4.91	0.82	
		IgM to *E. corrodens*	4.45 4.50 4.37	0.65 0.36 0.59	4.36	0.66	
		IgM to F. nucleatum*	3.15 3.72 3.26	0.70 0.97 0.92	3.29	0.88	
Albandar, 2002 49**	Radioimmunoassay	IL-1β*	21.5 20.9 17.5	19.6 19.6 19.6	23.6	19.6	pg/ml
Johnson, 1980 52	Radial immunodiffusion (RID)	Serum IgA*	141	69.3	213.3	118.4	mg/dl
		Serum IgM*	179.5	56.4	235.5	65	
		C3*	117.4	15.2	135.6	39.9	
		C4*	21.9	7.4	31.4	9.6	
Anil, 1990 50**	Radial immunodiffusion (RID)	CIC*	81.42	15.1	213.36	24.3	mg/dl
		IgG in CIC	48.46	20.91	26.12	20.13	
		IgM in CIC	26.81	9.29	17.41	8.52	
Albandar, 2002 49**	Radial immunodiffusion (RID)	Serum IgG*	16.9 15.9 16.4	3.7 3.7 3.6	16	3.7	mg/ml
		Serum IgA*	2.8 2.5 2.8	1.14 1.15 1.14	2.6	1.1	
		Serum IgM	2.4 2.4 2.2	0.9 1.04 1.03	2.2	1.02	
		IgG1*	10 9.9 9.5	2.64 2.63 2.64	9.7	2.6	
		IgG2*	4.8 4.4 5.1	1.64 1.62 1.60	4.5	1.65	
		IgG3	1 0.9 0.9	0.49 0.5 0.51	0.9	0.47	
		IgG4*	0.6 0.7 0.8	0.71 0.68 0.68	0.7	0.7	
Lehner, 1974 36**	Radial immunodiffusion (RID)	Serum IgG	C:1559.6 A:1785.3	220.5 318.03	C:1089.2 A:1451	219.2 467.38	mg/ml
		Serum IgA	C:296.3 A:297.5	103.14 109.52	C:185.4 A:242.5	70.4 90.12	
		Serum IgM	C:234.3 A:241.6	115.75 140.68	C:113.5 A:128.2	41.6 81.27	
Sandholm, 1983 45	Radial immunodiffusion (RID)	Serum α_2_M	2.91	0.85	NA		g/l
		Serum IgG	13.08	2.62			
		Serum IgA	2.66	1.39			
		Serum IgM	1.86	0.82			
		Total protein	71.16	8.3			
Spindler, 1985 54**	Electroimmunoassay	IgG/albumin ratio	0.5554	0.38	NA		
Sjödin, 1995 46	Lysis inhibition assay and gamma spectroscopy	Serum IgG	12.9	2.7	12.8	1.3	g/l
		Serum IgA	1.9	0.6	1.8	0.8	g/l
		Serum IgM	2.2	0.7	2.1	0.9	g/l
		ALP*	11.8	2.3	13	5.6	ucat/l
Dibart, 1998 51	Checkerboard immunoblotting	IgG1	(No numerical data presented)				NA
		IgG2					
		IgG3					
		IgG4					

^*^Values are higher in controls than in cases,**SD was calculated; *NA*, not applicable; *NR*, not reported; *ELISA*, enzyme-linked immunosorbent assay; *Aa*, *Aggregatibacter actinomycetemcomitans*; *ROS*, reactive oxygen species; *TRAP*, total radical-trapping antioxidant potential; *TBARs*, thiobarbituric acid-reactive substances; *RID*, radial immunodiffusion; *IgA*, immunoglobulin A; *GCF*, gingival crevicular fluid; *Interleukins*, IL-1β, IL-2, IL-6, IL-8, IL-10, IL-17 IL-12p40, IL-12p70; *GM-CSF*, granulocyte–macrophage colony-stimulating factor; *IP-10*, interferon γ-induced protein 10 kDa; *MCP-1*, monocyte chemoattractant protein-1; *MIP-1α*, macrophage inflammatory protein-1 alfa; *TNF-α*, tumour necrosis factor-alfa; *IFN-γ*, interferon-gamma; *RANKL*, receptor activator of NF-kappaB ligand; *OPG*, osteoprotegerin; *Matrix Metalloproteinase*, MMP-1, MMP-2, MMP-3, MMP-8, MMP-9, MMP-12, MMP-13; *IgG*, immunoglobulin G; *P. gingivalis*, *Porphyromonas gingivalis*; *B. fragilis*, *Bacteroides fragilis*; *IgM*, immunoglobulin M; *P. intermedia*, *Prevotella intermedi*; *C. rectus*, *Campylobacter rectus*; *E. corrodens*, *Eikenella corrodens*; *F. nucleatum*, *Fusobacterium nucleatum*; *C. ochracea*, *Capnocytophaga ochracea*; *CIC*, circulating immune complexes; *Immunoglobulins*, IgG1, IgG2, IgG3, IgG4; *Serum α2M*, serum alpha-2-macroglobulin; *ALP*, alkaline phosphatase; *LPS*, lipopolysaccharides; *ELP-α-PI complex*, ELP alfa proteinase inhibitor complex; *ELP*, elastase-like protease; *Differ. WBCC*, differential white blood cells; *HB*, hemoglobin; *MCV*, mean corpuscular volume; *LPK*, leukocytes; *ANC*, absolute neutrophil count; *TPK*, tyrosine protein kinase

### Characteristics of studies in whole blood

Three studies (two case–control and one cross-sectional) [44–46] from Turkey, Sweden, and Finland found that B-cells, CD3 + cells, CD4 + cells, CD8 + cells, and haemoglobin were all lower in cases than in controls. Lymphocyte counts were higher in cases in one study and lower in another, whereas mean corpuscular volume (MCV), leukocytes (LPK), absolute neutrophil count (ANC), and tyrosine-protein kinase (TPK) were higher in cases than controls. A meta-analysis was not possible due to the different biomarkers in whole blood used in the different studies.

### Characteristics of studies in plasma

Three studies (two case–control and one cohort) [27, 47, 48] from the USA and Germany analysed plasma samples for lipopolysaccharides (LPS), ELP-a-PI complex, and ELP content. As reported in Shaddox et al. study, LPS was significantly higher in cases than controls [27], and patients showed a significant reduction in LPS following treatment in Kalash et al. study [47]. ELP-a-PI complex was also statistically significant while ELP content was not [48]. A meta-analysis was not possible due to the different biomarkers in whole blood used in the different studies.

### Characteristics of studies in serum

**Fig. 2:**
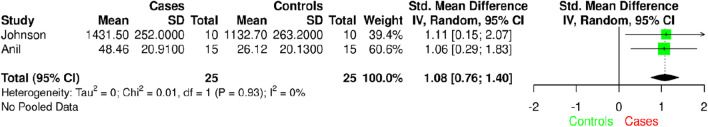
The meta-analysis of serum IgG in two studies

Several biomarkers were assessed in 13 studies (11 case–control and 2 cross-sectional) [28, 34, 36, 44–46, 49–55] from the USA, UK, Sweden, India, Turkey, Norway, and Finland. They are as follows: beta 2-microglobulin, IgG to *Aa*, IgG to *P. gingivalis*, IgG to *B. fragilis*, IgG to *P. intermedia*, IgG to *C. rectus*, IgG to *E. corrodens*, IgG to *F. nucleatum*, IgG to *C. ochracea,* IgA to *Aa*, IgA to *P. gingivalis*, IgA to *B. fragilis*, IgA to *P. intermedia*, IgA to *C. rectus*, IgA to *E. corrodens*, IgA to *F. nucleatum*, IgM to *Aa*, IgM to *P. gingivalis*, IgM *to B. fragilis*, IgM to *P. intermedia*, IgM to *C. rectus*, IgM to *E. corrodens*, IgM to *F. nucleatum*, IL-1β, C3, C4, IgG, IgA, IgM, IgG1, IgG2, IgG3, IgG4, α2M, protein, IgG/albumin ratio, and alkaline phosphatase ALP. Most investigated biomarkers were higher in cases than controls except for a few biomarkers that were lower in cases than controls as specified in Table 2. Six meta-analyses were performed involving the following molecules: IgG, IgM, IgG to *Aa*, IgG to *P. gingivalis*, IgM to *Aa*, and IgM to *P. gingivalis.* However, because of high heterogeneity (*I*^2^ > 75%), except for IgG, most meta-analyses should be considered inconclusive (Appendix 5). Only the meta-analysis of total serum IgG with low heterogeneity value revealed a significant increase in its levels in C/MIPs compared to controls (standardised mean difference: 1.08; 95% CI: 0.76, 1.40) (Fig. 2).

### Risk of bias analysis

All 28 studies were assessed for risk of bias. All cohort studies (4/4) and the majority of the case–control studies (19/20) revealed good quality whereas one case–control study had fair quality, and four cross-sectional studies had a high risk of bias (Appendix 6).

## Discussion

This review represents the first attempt to systematically assess biomarkers associated with the very unique phenotype of C/MIP periodontitis. The main findings are that there is a paucity of studies investigating this aspect and not many robust conclusions can be drawn. Although several reports suggest increased or decreased levels of specific inflammatory and tissue degradation markers in GCF, saliva, whole blood, serum, and plasma, meta-analysis was only possible for total IgG levels in serum. This analysis, based on only 2 papers, suggested increased total IgG levels in C/MIP cases compared with controls [50, 52]. Immunoglobulins (Ig) play a major role as part of humoral immunity by stimulating phagocytosis and eliminating microorganisms [56]. IgG is the most prevalent in human serum with periodontitis among the four other classes, IgA, IgM, IgE, and IgD [56], and that was consistent with the results of our meta-analysis and the literature.

Previous literature highlighted the host-microbial interactions and how the imbalance between them is essential for the occurrence of the disease and for determining the extent of the destruction [9, 40, 57]. Following colonisation by gram-negative bacteria including *A. actinomycetemcomitans*, *P. gingivalis*, and *Tannerella forsythia*, and the production of leukotoxins, endotoxins, collagenases, and proteases to cause bone resorption, the host responds by recruiting a significant amount of polymorphonuclear neutrophils (PMNs) including neutrophils, basophils, eosinophils in addition to monocytes, macrophages, and dendritic cells [58]. Particularly in the presence of neutrophils defects, periodontal destruction evolves aggressively resulting in rapid attachment and bone loss [59, 60]. The constant recruitment of host cells causes the oversecretion of several inflammatory mediators, including cytokines, tissue degradation markers, immunoglobulins, tumour markers, enzymes, and proteins [61]. Which can be found in larger quantities in C/MIP patients than in healthy controls, as listed in Table 2. In this context, biomarkers measuring the response to the microbial challenge could be valuable tools to corroborate the clinical findings and potentially have a diagnostic and prognostic added value.

The uniqueness of C/MIP lies in its rapidly-progressive nature and the irreversible periodontal damage caused at an early age and initially localised to the incisors and molars despite the minimal amounts of plaque, calculus, and marginal gingival inflammation [62], which suggests that microbes do not contribute solely to the severity of the disease [63]. The complexity of C/MIP makes it difficult to manage these cases especially since the plaque deposit is not the main etiological factor compared to other forms of periodontitis (formerly known as chronic periodontitis CP) [9]. In other types of periodontitis, maintaining good oral hygiene effectively reduces all the clinical parameters since the absence of bacteria is sufficient to arrest the disease [64]. Undoubtedly, clinical parameters help measure the current condition of the periodontium. However, they do not give a clear picture of the host-microbial interactions and stability or not of disease, especially for the very unique and poorly investigated C/MIP. Therefore molecular biomarkers could be beneficial, providing a diagnostic tool, which is relatively easy and painless to collect if present in saliva or GCF [65].

Treatment and long-term tooth retention may be challenging in C/MIP cases affecting young individuals [9, 40]. A treatment approach consisting of supra- and sub-gingival debridement with adjunctive systemic antibiotics was shown to assist in balancing the host immune responses and disease progression and significantly decrease disease biomarkers [40]. Surprisingly, in some studies, some biomarkers remained higher in cases than in controls even after receiving treatment [66].

Biomarkers have diagnostic and prognostic values as they are beneficial in understanding disease mechanisms and monitoring the host immune response before, during, and after the treatment [67]. Besides the biomarkers of C/MIP mentioned earlier, another set of biomarkers was significantly higher in patients with CP than in controls such as MCP-1, IL-6, MMP-8, macrophage inflammatory protein-1 alpha (MIP-1α), IL-1β, and Hb, and assessment of both salivary IL-6 and MMP-8 was used for early diagnosis [68]. In GCF, prostaglandin E2 (PGE2), aspartate aminotransferase (AST), IL-1β, IL-8, IL-10, neutrophil elastase (NE), osteocalcin and calprotectin, alkaline phosphatase (ALP), macroglobulins (alpha 2, beta 2), MMP-3, MMP-8, MMP-9 [69], MCP-1 [70], and deoxypyridinoline (DPD) have shown promise as biomarkers [71]. To the best of our knowledge, no systematic reviews/meta-analyses were conducted to comprehensively assess different periodontal biomarkers in the blood and serum of systemically healthy individuals. One review evaluated the blood cell count [72], while most existing reviews focused on specific biomarkers. Nonetheless, some potential biomarkers were noticed in the serum of patients with periodontitis, such as resistin [73], C-reactive protein [74], visfatin [75], oncostatin M [76], chemokine CXCL10 [77], and proprotein convertase subtilisin/kexin type 9 (PCSK9) [78]. In the blood, decreased total antioxidant status (TAS) [79] was observed in addition to the increased WBC and neutrophils and reduced erythrocytes and platelets [80]. While previous systematic reviews performed a meta-analysis of the diagnostic “accuracy” of biomarkers, meta-analysis for diagnostic accuracy could not be performed here, as none of the included papers reported the specificity and sensitivity, and only one study gave the diagnostic classification contingency table. Additionally, the paucity of data and high heterogeneity made it impossible to meta-analyse other biomarkers.

The four cohorts [37, 40, 42, 47] and nineteen case–control studies [27, 28, 32–34, 36, 38, 41, 43, 44, 46, 48–53, 55, 81] had a good quality for meeting NOS criteria in terms of selection, comparability, and exposure/outcome. However, one case control had a fair quality for not providing adequate definitions of stage III grade C and healthy controls [35]. The remaining cross-sectional studies did not have control groups to compare findings [39, 45, 54], failed to calculate and justify the sample size [39, 45, 54, 82], and did not control for confounding factors [39, 45, 54].

This review had several strengths including merging the biomarkers’ data of all the former classifications with the data of the 2017 new classification. The search was not limited to a specific language, as all relevant papers were included and translated if they were in a language other than English. Multiple main databases were searched to ensure that none of the relevant papers was missed unintentionally. Various types of samples were assessed to gain a comprehensive overview of existing biomarkers in the literature. The pre-specified age range was met as the included studies recruited subjects of 5–25 years of age. Although this might be considered a wide age range, it reflects the age range in most published studies. The authors of the papers with graphical representations of their data were contacted multiple times for the raw data. The main limitation of this review was the heterogeneity of the data among six studies that initially had the potential for meta-analysis of 18 biomarkers. Heterogeneity was very high in the 34 meta-analysis models performed, with the exception of the meta-analysis for IgG, so they were considered inconclusive. Another limitation was the lack of recent studies, as most studies (74%) were conducted more than 10 years ago, of which 55% were conducted before 1999. Also, when attempts were made to request raw data/full-text research for some studies, no contacts were found for some old publications and were therefore excluded for missing full-text. The graphs/plots were narratively described if the raw data was not received.

In conclusion, this review highlighted the existing gap in the literature regarding biomarkers of C/MIP and summarised what biomarkers had been investigated in saliva, GCF, blood, plasma, and serum to date. The results emphasise the importance of conducting future observational studies to identify reliable biomarkers that could be useful adjunctive diagnostic tools and/or could accurately predict the likelihood of developing C/MIP before it occurs. This will contribute to prevention/early diagnosis, better treatment outcome, and maintenance of the quality of life. More robust research studies should be conducted in this area, ideally investigating large cohorts of young individuals affected by C/MIP and reporting data on biomarkers that could have clinical utility and could potentially be used for larger meta-analyses.

## Supplementary Information

Below is the link to the electronic supplementary material.

Supplementary file1 (DOCX 33 KB)

Supplementary file2 (DOCX 17 KB)

Supplementary file3 (DOCX 103 KB)

Supplementary file4 (DOCX 1214 KB)

Supplementary file5 (DOCX 553 KB)

Supplementary file6 (DOCX 39 KB)

## Data Availability

The data supporting this study’s findings are available on request from the corresponding author.

## References

[1] Wara-aswapati N, Howell TH, Needleman HL, Karimbux N (1999). Periodontitis in the child and adolescent. ASDC J Dent Child.

[2] Zhang L, Henson BS (2000). Camargo PM and Wong DT (2009) The clinical value of salivary biomarkers for periodontal disease. Periodontol.

[3] Velsko IM, Harrison P, Chalmers N, Barb J, Huang H, Aukhil I, Shaddox L (2020). Grade C molar-incisor pattern periodontitis subgingival microbial profile before and after treatment. J Oral Microbiol.

[4] Shaddox LM, Huang H, Lin T, Hou W, Harrison PL, Aukhil I, Walker CB, Klepac-Ceraj V, Paster BJ (2012). Microbiological characterization in children with aggressive periodontitis. J Dent Res.

[5] Meusel DR, Ramacciato JC, Motta RH, Brito Júnior RB, Flório FM (2015). Impact of the severity of chronic periodontal disease on quality of life. J Oral Sci.

[6] Allin N, Cruz-Almeida Y, Velsko I, Vovk A, Hovemcamp N, Harrison P, Huang H, Aukhil I, Wallet SM, Shaddox LM (2016). Inflammatory response influences treatment of localized aggressive periodontitis. J Dent Res.

[7] Papapanou PN, Sanz M, Buduneli N, Dietrich T, Feres M, Fine DH, Flemmig TF, Garcia R, Giannobile WV, Graziani F, Greenwell H, Herrera D, Kao RT, Kebschull M, Kinane DF, Kirkwood KL, Kocher T, Kornman KS, Kumar PS, Loos BG, Machtei E, Meng H, Mombelli A, Needleman I, Offenbacher S, Seymour GJ, Teles R, Tonetti MS (2018). Periodontitis: consensus report of workgroup 2 of the 2017 World Workshop on the classification of periodontal and peri-implant diseases and conditions. J Clin Periodontol.

[8] Yoshida A, Bouziane A, Erraji S, Lakhdar L, Rhissassi M, Miyazaki H, Ansai T, Iwasaki M, Ennibi O (2021). Etiology of aggressive periodontitis in individuals of African descent. Jpn Dent Sci Rev.

[9] Albandar JM (2000). (2014) Aggressive periodontitis: case definition and diagnostic criteria. Periodontol.

[10] Miller K, Treloar T, Guelmann M, Rody WJ, Jr. and Shaddox LM (2018) Clinical characteristics of localized aggressive periodontitis in primary dentition. J Clin Pediatr Dent 42:95–102. 10.17796/1053-4628-42.2.3 10.17796/1053-4628-42.2.3 PMC5906128 29087795

[11] Llanos AH, Silva CGB, Ichimura KT, Rebeis ES, Giudicissi M, Romano MM, Saraiva L (2018). Impact of aggressive periodontitis and chronic periodontitis on oral health-related quality of life. Braz Oral Res.

[12] Prakasam A, Elavarasu SS, Natarajan RK (2012). Antibiotics in the management of aggressive periodontitis. J Pharm Bioallied Sci.

[13] Saygun I, Nizam N, Keskiner I, Bal V, Kubar A, Açıkel C, Serdar M, Slots J (2011). Salivary infectious agents and periodontal disease status. J Periodontal Res.

[14] Mishra S, Gazala MP, Rahman W (2022). Clinical and diagnostic significance of blood leukocyte ratios in young patients with stage III grade C periodontitis. Acta Odontol Scand.

[15] Patil PB, Patil BR (2011). Saliva: a diagnostic biomarker of periodontal diseases. J Indian Soc Periodontol.

[16] Arias-Bujanda N, Regueira-Iglesias A, Balsa-Castro C, Nibali L, Donos N, Tomás I (2020). Accuracy of single molecular biomarkers in saliva for the diagnosis of periodontitis: a systematic review and meta-analysis. J Clin Periodontol.

[17] Fatima T, Khurshid Z, Rehman A, Imran E, Srivastava KC, Shrivastava D (2021) Gingival crevicular fluid (GCF): a diagnostic tool for the detection of periodontal Health and diseases. Molecules 26. 10.3390/molecules26051208 10.3390/molecules26051208 PMC7956529 33668185

[18] Giannobile WV, Beikler T, Kinney JS, Ramseier CA (2000). Morelli T and Wong DT (2009) Saliva as a diagnostic tool for periodontal disease: current state and future directions. Periodontol.

[19] Subbarao KC, Nattuthurai GS, Sundararajan SK, Sujith I, Joseph J, Syedshah YP (2019). Gingival crevicular fluid: an overview. J Pharm Bioallied Sci.

[20] de Lima CL, Acevedo AC, Grisi DC, Taba M, Guerra E, De Luca CG (2016). Host-derived salivary biomarkers in diagnosing periodontal disease: systematic review and meta-analysis. J Clin Periodontol.

[21] Schenkein HA, Koertge TE, Brooks CN, Sabatini R, Purkall DE, Tew JG (2010). IL-17 in sera from patients with aggressive periodontitis. J Dent Res.

[22] Stefaniuk P, Szymczyk A, Podhorecka M (2020). The neutrophil to lymphocyte and lymphocyte to monocyte ratios as new prognostic factors in hematological malignancies - a narrative review. Cancer Manag Res.

[23] Wang H, Luo Z, Lei L, Sun Z, Zhou M, Dan H, Zeng X, Chen Q (2013). Interaction between oral lichen planus and chronic periodontitis with Th17-associated cytokines in serum. Inflammation.

[24] Andreu R, Santos-Del-Riego S and Payri F (2021) Serum inflammatory and prooxidant marker levels in different periodontal disease stages. Healthcare (Basel) 9. 10.3390/healthcare9081070 10.3390/healthcare9081070 PMC8391602 34442206

[25] Machado V, Botelho J, Escalda C, Hussain SB, Luthra S, Mascarenhas P, Orlandi M, Mendes JJ, D’Aiuto F (2021). Serum C-reactive protein and periodontitis: a systematic review and meta-analysis. Front Immunol.

[26] Bramer WM, Giustini D, de Jonge GB, Holland L, Bekhuis T (2016). De-duplication of database search results for systematic reviews in EndNote. J Med Libr Assoc.

[27] Shaddox LM, Wiedey J, Calderon NL, Magnusson I, Bimstein E, Bidwell JA, Zapert EF, Aukhil I, Wallet SM (2011). Local inflammatory markers and systemic endotoxin in aggressive periodontitis. J Dent Res.

[28] Albandar JM, DeNardin AM, Adesanya MR, Diehl SR, Winn DM (2001). Associations between serum antibody levels to periodontal pathogens and early-onset periodontitis. J Periodontol.

[29] Ebert MHaPCaTFaDD (2019) dmetar: companion R package for the guide ‘doing meta-analysis in R’. http://dmetar.protectlab.org/. Accessed Acces Date

[30] Balduzzi S, Rücker G, Schwarzer G (2019). How to perform a meta-analysis with R: a practical tutorial. Evid Based Ment Health.

[31] Viechtbauer W (2005). Bias and efficiency of meta-analytic variance estimators in the random-effects model. J Educ Behav Stat.

[32] Acquier AB, De Couto Pita AK, Busch L, Sanchez GA (2017). Parameters of oxidative stress in saliva from patients with aggressive and chronic periodontitis. Redox Rep:Commun Free Radic Res.

[33] Acquier AB, Pita AKDC, Busch L, Sanchez GA (2015). Comparison of salivary levels of mucin and amylase and their relation with clinical parameters obtained from patients with aggressive and chronic periodontal disease. J Appl Oral Sci: revista FOB.

[34] Akalin FA, Bulut S, Yavuzyilmaz E (1993). Beta 2-microglobulin levels in serum and saliva of patients with juvenile periodontitis. J Nihon Univ Sch Dent.

[35] Fine DH, Furgang D, McKiernan M, Rubin M (2013). Can salivary activity predict periodontal breakdown in A. actinomycetemcomitans infected adolescents?. Arch Oral Biol.

[36] Lehner T, Wilton JM, Ivanyi L, Manson JD (1974). Immunological aspects of juvenile periodontitis (periodontosis). J Periodontal Res.

[37] Albandar JM, Kingman A, Lamster IB (1998). Crevicular fluid level of beta-glucuronidase in relation to clinical periodontal parameters and putative periodontal pathogens in early-onset periodontitis. J Clin Periodontol.

[38] Alfant B, Shaddox LM, Tobler J, Magnusson I, Aukhil I, Walker C (2008). Matrix metalloproteinase levels in children with aggressive periodontitis. J Periodontol.

[39] Bartova J, Krejsa O, Sirova M, Tlaskalova H, Prochazkova J, Duskova J (1995). Local antibodies and cytokine responses in crevicular fluid of patients with juvenile periodontitis. Adv Exp Med Biol.

[40] Branco-de-Almeida LS, Cruz-Almeida Y, Gonzalez-Marrero Y, Kudsi R, de Oliveira ICV, Dolia B, Huang H, Aukhil I, Harrison P, Shaddox LM (2021). Treatment of localized aggressive periodontitis alters local host immunoinflammatory profiles: a long-term evaluation. J Clin Periodontol.

[41] Friedman SA, Mandel ID, Herrera MS (1983). Lysozyme and lactoferrin quantitation in the crevicular fluid. J Periodontol.

[42] Goncalves PF, Huang H, McAninley S, Alfant B, Harrison P, Aukhil I, Walker C, Shaddox LM (2013). Periodontal treatment reduces matrix metalloproteinase levels in localized aggressive periodontitis. J Periodontol.

[43] Monteiro MF, Casati MZ, Sallum EA, Silverio KG, Nociti-Jr FH, Casarin RCV (2022). The familial trend of the local inflammatory response in periodontal disease. Oral Dis.

[44] Celenligil H, Ebersole JL (1998). Analysis of serum antibody responses to periodontopathogens in early-onset periodontitis patients from different geographical locations. J Clin Periodontol.

[45] Sandholm L, Saxen L (1983). Concentrations of serum protease inhibitors and immunoglobulins in juvenile periodontitis. J Periodontal Res.

[46] Sjodin B, Arnrup K, Matsson L, Wranne L, Carlsson J, Hanstrom L (1995). Periodontal and systemic findings in children with marginal bone loss in the primary dentition. J Clin Periodontol.

[47] Kalash D, Vovk A, Huang H, Aukhil I, Wallet SM, Shaddox LM (2015). Influence of periodontal therapy on systemic lipopolysaccharides in children with localized aggressive periodontitis. Pediatr Dent.

[48] Zafiropoulos GG, Eldanassouri N, Flores-de-Jacoby L, Havemann K (1987). Determination of the ELP (elastase-like proteinase) plasma levels in patients with rapidly advancing and with juvenile periodontitis. Deutsche zahnarztliche Zeitschrift.

[49] Albandar JM, DeNardin AM, Adesanya MR, Winn DM, Diehl SR (2002). Associations of serum concentrations of IgG, IgA, IgM and interleukin-1beta with early-onset periodontitis classification and race. J Clin Periodontol.

[50] Anil S, Remani P, Ankathil R, Vijayakumar T (1990). Circulating immune complexes in localised juvenile periodontitis. Singapore Dent J.

[51] Dibart S, Eftimiadi C, Socransky S, Taubman MA, Van Dyke TE (1998). Rapid evaluation of serum and gingival crevicular fluid immunoglobulin G subclass antibody levels in patients with early-onset periodontitis using checkerboard immunoblotting. Oral Microbiol Immunol.

[52] Johnson RJ, Matthews JL, Stone MJ, Hurt WC, Newman JT (1980). Immunopathology of periodontal disease. I. Immunologic profiles in periodontitis and juvenile periodontitis. J Periodontol.

[53] Schenck K, Porter SR, Tollefsen T, Johansen JR, Scully C (1989). Serum levels of antibodies against Actinobacillus actinomycetemcomitans in various forms of human periodontitis. Acta Odontol Scand.

[54] Spindler SJ, Thompson JJ, Yukna RA, Costales AD (1986). Juvenile periodontitis. I. Demonstration of local immunoglobulin synthesis. J Periodontol.

[55] Unsal BT, Ozcan G, Balos K, Mevsim G (1996). Serum antibodies to Actinobacillus actinomycetemcomitans and Porphyromonas gingivalis in juvenile periodontitis and adult periodontitis (part I). J Marmara Univ Dent Fac.

[56] Kulshrestha D, Siddeshappa S, Biswas J (2013). Role of immunoglobulin G and A in periodontitis: a review. Jof Pure Appl Microbiol.

[57] Nibali L (2015). Aggressive periodontitis: microbes and host response, who to blame?. Virulence.

[58] Kantarci A, Oyaizu K, Van Dyke TE (2003). Neutrophil-mediated tissue injury in periodontal disease pathogenesis: findings from localized aggressive periodontitis. J Periodontol.

[59] Bhansali RS, Yeltiwar RK, Bhat K (2017). Evaluation of peripheral neutrophil functions in aggressive periodontitis patients and their family members in Indian population: an assessment of neutrophil chemotaxis, phagocytosis, and microbicidal activity. J Indian Soc Periodontol.

[60] Meng H, Xu L, Li Q (2000). Han J and Zhao Y (2007) Determinants of host susceptibility in aggressive periodontitis. Periodontol.

[61] Kornman KS (2008). Mapping the pathogenesis of periodontitis: a new look. J Periodontol.

[62] Niklaus Lang PMB, Cullinan M, Jeffcoat M, Mombelli A, Murakami S, Page R, Papapanou P, Tonetti M, Van Dyke T (1999). Consensus report: aggressive periodontitis. Ann Periodontol.

[63] Mani A, James R, Mani S (2018) Etiology and pathogenesis of aggressive periodontitis: a mini review. Galore Int J Health Sci Res 3(2):4–8

[64] Lertpimonchai A, Rattanasiri S, Arj-Ong Vallibhakara S, Attia J, Thakkinstian A (2017). The association between oral hygiene and periodontitis: a systematic review and meta-analysis. Int Dent J.

[65] Ji S, Choi Y (2015). Point-of-care diagnosis of periodontitis using saliva: technically feasible but still a challenge. Front Cell Infect Microbiol.

[66] Trombelli L, Scapoli C, Tatakis DN, Minenna L (2006). Modulation of clinical expression of plaque-induced gingivitis: response in aggressive periodontitis subjects. J Clin Periodontol.

[67] Bhuyan R, Bhuyan SK, Mohanty JN, Das S, Juliana N and Juliana IF (2022) Periodontitis and its inflammatory changes linked to various systemic diseases: a review of its underlying mechanisms. Biomedicines 10. 10.3390/biomedicines10102659 10.3390/biomedicines10102659 PMC9599402 36289921

[68] Cafiero C, Spagnuolo G, Marenzi G, Martuscelli R, Colamaio M and Leuci S (2021) Predictive periodontitis: the most promising salivary biomarkers for early diagnosis of periodontitis. J Clin Med 10. 10.3390/jcm10071488 10.3390/jcm10071488 PMC8038382 33916672

[69] Gupta S, Chhina S, Arora SA (2018). A systematic review of biomarkers of gingival crevicular fluid: their predictive role in diagnosis of periodontal disease status. J Oral Biol Craniofac Res.

[70] Gupta M, Chaturvedi R, Jain A (2013). Role of monocyte chemoattractant protein-1 (MCP-1) as an immune-diagnostic biomarker in the pathogenesis of chronic periodontal disease. Cytokine.

[71] Syed S, Kankara VR, Pathakota KR, Krishnan P, Mishra A (2020). Evaluation of deoxypyridinoline levels in gingival crevicular fluid and serum as alveolar bone loss biomarker in patients with periodontitis. J Indian Soc Periodontol.

[72] Almășan O, Leucuța DC and Hedeșiu M (2022) Blood cell count inflammatory markers as prognostic indicators of periodontitis: a systematic review and meta-analysis. J Pers Med 12. 10.3390/jpm12060992 10.3390/jpm12060992 PMC9225277 35743775

[73] Akram Z, Rahim ZH, Taiyeb-Ali TB, Shahdan MS, Baharuddin NA, Vaithilingam RD, Safii SH (2017). Resistin as potential biomarker for chronic periodontitis: a systematic review and meta-analysis. Arch Oral Biol.

[74] Pradeep AR, Manjunath RG, Kathariya R (2010). Progressive periodontal disease has a simultaneous incremental elevation of gingival crevicular fluid and serum CRP levels. J Investig Clin Dent.

[75] Türer ÇC, Balli U, Güven B, Çetinkaya B, Keleş G (2016). Visfatin levels in gingival crevicular fluid and serum before and after non-surgical treatment for periodontal diseases. J Oral Sci.

[76] Thorat M, Pradeep AR, Garg G (2010). Correlation of levels of oncostatin M cytokine in crevicular fluid and serum in periodontal disease. Int J Oral Sci.

[77] Aldahlawi S, Youssef AR, Shahabuddin S (2018). Evaluation of chemokine CXCL10 in human gingival crevicular fluid, saliva, and serum as periodontitis biomarker. J Inflamm Res.

[78] Tabeta K, Hosojima M, Nakajima M, Miyauchi S, Miyazawa H, Takahashi N, Matsuda Y, Sugita N, Komatsu Y, Sato K, Ishikawa T, Akiishi K, Yamazaki K, Kato K, Saito A, Yoshie H (2018). Increased serum PCSK9, a potential biomarker to screen for periodontitis, and decreased total bilirubin associated with probing depth in a Japanese community survey. J Periodontal Res.

[79] Konopka T, Król K, Kopeć W, Gerber H (2007). Total antioxidant status and 8-hydroxy-2'-deoxyguanosine levels in gingival and peripheral blood of periodontitis patients. Arch Immunol Ther Exp (Warsz).

[80] Botelho J, Machado V, Hussain SB, Zehra SA, Proença L, Orlandi M, Mendes JJ, D'Aiuto F (2021). Periodontitis and circulating blood cell profiles: a systematic review and meta-analysis. Exp Hematol.

[81] Celenligil H, Kansu E, Eratalay K (1990). Juvenile and rapidly progressive periodontitis. Peripheral blood lymphocyte subpopulations. J Clin Periodontol.

[82] Tavakoli TT, Gholami F, Huang H, Gonçalves PF, Villasante-Tezanos A, Aukhil I, de Oliveira RCG, Hovencamp N, Wallet S, Ioannidou E, Shaddox LM (2022). Gender differences in immunological response of African-American juveniles with grade C molar incisor pattern periodontitis. J Periodontol.

