# Supplementary material for: Biomarkers for diagnosis of stage III, grade C with molar incisor pattern periodontitis in children and young adults: a systematic review and meta-analysis
Source: Clin Oral Investig. 2023 Aug 3;27(9):4929–55. doi: 10.1007/s00784-023-05169-x (PMC10492694; doi:10.1007/s00784-023-05169-x)
Supplement: Supplementary file 2 — Supplementary file2 (DOCX 17 KB) [file 784_2023_5169_MOESM2_ESM.docx]

**Appendix1: Search strings for each database**

| Database | Search strings |
| --- | --- |
| [Embase](about:blank)  (via Ovid) | 1. periodontal disease* 2. periodontitis 3. aggressive periodontitis 4. juvenile periodontitis 5. Grade C periodontitis 6. early onset periodontitis 7. Prepubertal periodontitis 8. Rapidly Progressive Periodontitis 9. 1 OR 2 OR 3 OR 4 OR 5 OR 6 OR 7 OR 8 10. salivary biomarker* 11. salivary marker* 12. biomarker* 13. marker* 14. cytokine* 15. interleukin* 16. Immunoglobulin* 17. Matrix metalloproteinase* 18. MMP* 19. saliva* 20. gingival crevicular fluid 21. GCF 22. blood 23. serum 24. 10 OR 11 OR 12 OR 13 OR 14 OR 15 OR 16 OR 17 OR 18 OR 19 OR 20 OR 21 OR 22 OR 23 25. child* 26. adolescent* 27. pre-school 28. preschool* 29. young* 30. 25 OR 26 OR 27 OR 28 OR 29 31. 9 AND 24 32. 30 AND 31 |
| [Web of Science](about:blank) | (“periodontal disease” OR “periodontitis” OR “aggressive periodontitis” OR “juvenile periodontitis” OR “Grade C periodontitis” OR “early onset periodontitis” OR “Prepubertal periodontitis” OR “Rapidly Progressive Periodontitis” ) AND (“salivary biomarker*” OR “salivary marker*” OR “biomarker*” OR “marker*” OR “cytokine*” OR “interleukin*” OR “Immunoglobulin*” OR “Matrix metalloproteinase*” OR “MMP*” OR “saliva*” OR “gingival crevicular fluid” OR “GCF” OR “blood” OR “serum”) AND (child* OR adolescent* OR pre-school OR preschool* OR young*) |
| [Medline](about:blank)  (Via Pubmed) | (“periodontal disease” OR “periodontitis” OR “aggressive periodontitis” OR “juvenile periodontitis” OR “Grade C periodontitis” OR “early onset periodontitis” OR “Prepubertal periodontitis” OR “Rapidly Progressive Periodontitis” ) AND (“salivary biomarker*” OR “salivary marker*” OR “biomarker*” OR “marker*” OR “cytokine*” OR “interleukin*” OR “Immunoglobulin*” OR “Matrix metalloproteinase*” OR “MMP*” OR “saliva*” OR “gingival crevicular fluid” OR “GCF” OR “blood” OR “serum”) AND (child* OR adolescent* OR pre-school OR preschool* OR young*) |
| [Virtual Health Library](about:blank) | (“periodontal disease” OR “periodontitis” OR “aggressive periodontitis” OR “juvenile periodontitis” OR “Grade C periodontitis” OR “early onset periodontitis” OR “Prepubertal periodontitis” OR “Rapidly Progressive Periodontitis”) |
| Scopus | (“periodontal disease” OR “periodontitis” OR “aggressive periodontitis” OR “juvenile periodontitis” OR “Grade C periodontitis” OR “early onset periodontitis” OR “Prepubertal periodontitis” OR “Rapidly Progressive Periodontitis” ) AND (“salivary biomarker*” OR “salivary marker*” OR “biomarker*” OR “marker*” OR “cytokine*” OR “interleukin*” OR “Immunoglobulin*” OR “Matrix metalloproteinase*” OR “MMP*” OR “saliva*” OR “gingival crevicular fluid” OR “GCF” OR “blood” OR “serum”) AND (“child*” OR “adolescent*” OR “pre-school” OR “preschool*” OR “young*”) |
| ProQuest | noft(periodontal* OR periodontitis) AND noft(biomarker* OR marker* OR cytokine* OR interleukin* OR Immunoglobulin* OR Matrix metalloproteinase OR MMP OR saliva* OR gingival crevicular fluid OR GCF OR blood OR serum) AND noft(child* OR adolescent OR pre-school OR preschool OR young*) |
