# Supplementary material for: Biomarkers for diagnosis of stage III, grade C with molar incisor pattern periodontitis in children and young adults: a systematic review and meta-analysis
Source: Clin Oral Investig. 2023 Aug 3;27(9):4929–55. doi: 10.1007/s00784-023-05169-x (PMC10492694; doi:10.1007/s00784-023-05169-x)
Supplement: Supplementary file 3 — Supplementary file3 (DOCX 103 KB) [file 784_2023_5169_MOESM3_ESM.docx]

**Appendix 3. Screening decision of 437 articles and reason for including/excluding each article.**

| # | Author and year of publication | Title | Decision | Reason |
| --- | --- | --- | --- | --- |
|  | Akalin 1993 | Beta 2-Microglobulin levels in serum and saliva of patients with juvenile periodontitis | Include | Met the criteria |
|  | Bartova 2000 | Th1 and Th2 cytokine profile in patients with early onset periodontitis and their healthy siblings | Exclude | <10 subjects |
|  | Branco-de-Almeida 2020 | Treatment of localized aggressive periodontitis alters local host immunoinflammatory profiles: A long-term evaluation | Include | Met the criteria |
|  | Bresolin 2014 | Effectiveness of periodontal treatment on the improvement of inflammatory markers in children | Exclude | Not Stage III Grade C |
|  | Celenligil 1990 | Juvenile and rapidly progressive periodontitis. Peripheral blood lymphocyte subpopulations | Include | Met the criteria |
|  | Chaudhari 2016 | Association of Interleukin-17 polymorphism (-197G/A) in chronic and localized aggressive periodontitis | Exclude | Genetic profile |
|  | Chen 2018 | Analysis of metabolic profiles of generalized aggressive periodontitis | Exclude | Age > 25 |
|  | Chung 1986 | Antibodies to *Actinobacillus actinomycetemcomitans* in a Korean population | Exclude | Not Stage III Grade C |
|  | Chung 2003 | Immunoglobulin G profiles in different forms of periodontitis | Exclude | Age > 25 |
|  | Cifcibasi 2015 | Evaluation of Local and Systemic Levels of Interleukin-17, Interleukin-23, and Myeloperoxidase in Response to Periodontal Therapy in Patients with Generalized Aggressive Periodontitis | Exclude | Age > 25 |
|  | Cole 1995 | Humoral immunity to commensal oral bacteria: quantitation, specificity and avidity of serum IgG and IgM antibodies reactive with *Actinobacillus actinomycetemcomitans* in children | Exclude | <10 subjects |
|  | Dibart 1998 | Rapid evaluation of serum and gingival crevicular fluid immunoglobulin G subclass antibody levels in patients with early-onset periodontitis using checkerboard immunoblotting | Include | Met the criteria only for the LAgP group |
|  | Drugarin 1998 | Proinflammatory cytokines production and PMN-elastase release from activated PMN cells in the periodontal disease | Exclude | Age > 25 |
|  | Ebersole 1982 | Human immune responses to oral micro-organisms. I. Association of localized juvenile periodontitis (LJP) with serum antibody responses to *Actinobacillus actinomycetemcomitans* | Exclude | Age > 25 |
|  | Elabdeen 2013 | Ratio of pro-resolving and pro-inflammatory lipid mediator precursors as potential markers for aggressive periodontitis | Exclude | Age > 25 |
|  | Ertugrul 2013 | Comparison of CCL28, interleukin-8, interleukin-1beta and tumor necrosis factor-alpha in subjects with gingivitis, chronic periodontitis and generalized aggressive periodontitis | Exclude | Age > 25 |
|  | Fine 2009 | Macrophage inflammatory protein-1alpha: a salivary biomarker of bone loss in a longitudinal cohort study of children at risk for aggressive periodontal disease? | Exclude | <10 subjects |
|  | Fujita 2005 | CD38 expression in neutrophils from patients with localized aggressive periodontitis | Exclude | Age > 25 |
|  | Gaddale 2016 | Changes in cellular and molecular components of peripheral blood in patients with generalized aggressive periodontitis | Exclude | Age > 25 |
|  | Gainet 1999 | Neutrophil dysfunctions, IL-8, and soluble L-selectin plasma levels in rapidly progressive versus adult and localized juvenile periodontitis: Variations according to disease severity and microbial flora | Exclude | Unclear age |
|  | Gonzales 2011 | Production of interleukin-13 is influenced by the interleukin-4 -34TT and -590TT genotype in patients with aggressive periodontitis | Exclude | Age > 25 |
|  | Goyal 2014 | Comparative evaluation of serum C-reactive protein levels in chronic and aggressive periodontitis patients and association with periodontal disease severity | Exclude | Age > 25 |
|  | Gregory 1992 | Immunoglobulin-degrading enzymes in localized juvenile periodontitis | Exclude | Age > 25 |
|  | GuÃ­zar-Mendoza 2018 | Correlation between interleukin-1[beta] and matrix metalloproteinase-1 levels in crevicular fluid with a proposed periodontal disease index in children | Exclude | Not Stage III Grade C |
|  | Gunpinar 2017 | Gingival crevicular fluid levels of monocyte chemoattractant protein-1 in patients with aggressive periodontitis | Exclude | Unclear age |
|  | Gurkan 2007 | Matrix metalloproteinase -2, -9, and -12 gene polymorphisms in generalized aggressive periodontitis | Exclude | Age > 25 |
|  | Hormia 1993 | Increased rate of salivary epidermal growth factor secretion in patients with juvenile periodontitis | Exclude | Age > 25 |
|  | Katarkar 2015 | Telomerase expression in individuals with chronic and aggressive periodontitis | Exclude | Age > 25 |
|  | Kinane 1989 | Depressed helper-to-suppressor T-cell ratios in early-onset forms of periodontal disease | Exclude | <10 subjects |
|  | Leino 1994 | Diacylglycerol in peripheral blood neutrophils from patients with localized juvenile periodontitis | Exclude | Age > 25 |
|  | Lima 2011 | Aggressive and chronic periodontitis correlate with distinct cellular sources of key immunoregulatory cytokines | Exclude | Age > 25 |
|  | Ling 1993 | Titer and subclass distribution of serum IgG antibody reactive with *Actinobacillus actinomycetemcomitans* in localized juvenile periodontitis | Exclude | Age > 25 |
|  | Lira-Junior 2017 | Salivary and Serum Markers Related to Innate Immunity in Generalized Aggressive Periodontitis | Exclude | Age > 25 |
|  | Liu 2009 | Elevated plasma calcifediol is associated with aggressive periodontitis | Exclude | Age > 25 |
|  | Lu 2008 | [Analysis of short chain fatty acids in gingival crevicular fluid of patients with aggressive periodontitis] | Exclude | Age > 25 |
|  | Mishra 2021 | Clinical and diagnostic significance of blood leukocyte ratios in young patients with stage III grade C periodontitis | Exclude | Age > 25 |
|  | Monteiro 2022 | The familial trend of the local inflammatory response in periodontal disease | Include | Met the criteria |
|  | Narendra 2018 | Superoxide Dismutase, Uric Acid, Total Antioxidant Status, and Lipid Peroxidation Assay in Chronic and Aggressive Periodontitis Patients | Exclude | Age > 25 |
|  | Nizam 2014 | Serum and salivary matrix metalloproteinases, neutrophil elastase, myeloperoxidase in patients with chronic or aggressive periodontitis | Exclude | Age > 25 |
|  | Offenbacher 1984 | Crevicular fluid prostaglandin E levels as a measure of the periodontal disease status of adult and juvenile periodontitis patients | Exclude | Unclear age |
|  | Ozmeric 2002 | Level of neopterin, a marker of immune cell activation in gingival crevicular fluid, saliva, and urine in patients with aggressive periodontitis | Exclude | Age > 25 |
|  | Pai B 2019 | Correlations between Interleukin-33 and -1alpha Levels in Gingival Crevicular Fluid and Plasma in Patients with Chronic or Aggressive Periodontitis and Disease-free Subjects | Exclude | Age > 25 |
|  | Gorgun 2017 | IL-6 and IL-10 gene polymorphisms in patients with aggressive periodontitis: effects on GCF, serum and clinic parameters | Exclude | Age > 25 |
|  | Salzberg 2006 | C-reactive protein levels in patients with aggressive periodontitis | Exclude | Age > 25 |
|  | Sanchez-Hernandez 2011 | IL-12 and IL-18 levels in serum and gingival tissue in aggressive and chronic periodontitis | Exclude | Age > 25 |
|  | S and holm 1984 | Salivary immunoglobulins in patients with juvenile periodontitis and their healthy siblings | Exclude | Age > 25 |
|  | Saraiva 2014 | IgG sera levels against a subset of periodontopathogens and severity of disease in aggressive periodontitis patients: a cross-sectional study of selected pocket sites | Exclude | Unclear age |
|  | Schenck 1989 | Serum levels of antibodies against *Actinobacillus actinomycetemcomitans* in various forms of human periodontitis | Include | Met the criteria |
|  | Schenkein 2010 | IL-17 in sera from patients with aggressive periodontitis | Exclude | Age > 25 |
|  | Shaddox 2011 | Local inflammatory markers and systemic endotoxin in aggressive periodontitis | Include | Met the criteria |
|  | Shankar 2018 | Assessment of serum ceruloplasmin levels in gingivitis, chronic and aggressive periodontitis patients- a clinico-biochemical study | Exclude | Age > 25 |
|  | Shapira 1994 | The secretion of PGE2, IL-1 beta, IL-6, and TNF alpha by adherent mononuclear cells from early onset periodontitis patients | Exclude | Age > 25 |
|  | Sun 2008 | Levels of interleukin-1beta and tumor necrosis factor-alpha in patients with aggressive periodontitis | Exclude | Age > 25 |
|  | Teodorescu 2019 | Assessment of Salivary Levels of RANKL and OPG in Aggressive versus Chronic Periodontitis | Exclude | Age > 25 |
|  | Toyman 2015 | Evaluation of gingival crevicular fluid levels of tissue plasminogen activator, plasminogen activator inhibitor 2, matrix metalloproteinase-3 and interleukin 1-beta in patients with different periodontal diseases | Exclude | Age > 25 |
|  | Tripathi 2018 | Assessment of Lipid Peroxidation Levels and Total Antioxidant Status in Chronic and Aggressive Periodontitis Patients: An in vivo Study | Exclude | Age > 25 |
|  | Turkoglu 2017 | Gingival crevicular fluid and serum hCAP18/LL-37 levels in generalized aggressive periodontitis | Exclude | Age > 25 |
|  | Waldrop 1981 | IgG and IgG subclasses in human periodontosis (juvenile periodontitis). Serum concentrations | Exclude | <10 subjects |
|  | Whitney 1992 | Serum immunoglobulin G antibody to *Porphyromonas gingivalis* in rapidly progressive periodontitis: titer, avidity, and subclass distribution | Exclude | Age > 25 |
|  | Wilson 1992 | Immunoglobulin G subclass response of localized juvenile periodontitis patients to *Actinobacillus actinomycetemcomitans* Y4 lipopolysaccharide | Exclude | Age > 25 |
|  | Wilson 1995 | Immunoglobulin G subclass response of juvenile periodontitis subjects to principal outer membrane proteins of *Actinobacillus actinomycetemcomitans* | Exclude | Age > 25 |
|  | Wohlfeil 2012 | Increased systemic elastase and C-reactive protein in aggressive periodontitis (CLOI-D-00160R2) | Exclude | Age > 25 |
|  | Acquier 2017 | Parameters of oxidative stress in saliva from patients with aggressive and chronic periodontitis | Include | Met the criteria |
|  | Acquier 2015 | Comparison of salivary levels of mucin and amylase and their relation with clinical parameters obtained from patients with aggressive and chronic periodontal disease | Include | Met the criteria |
|  | Agarkov 2020 | Dynamics of Humoral Immunity in Gingival Fluid during Combined Immunotherapy in Pediatric Patients with Exacerbation of Chronic Granulomatous Periodontitis | Exclude | Not Stage III Grade C |
|  | Agarkov 2018 | [The prognostication of development of peri-apical abscess under chronic periodontitis by blood parameters and cell immunity in children] | Exclude | Not Stage III Grade C |
|  | Aguilera-Galaviz 2019 | Nutritional and Oral Health Conditions in High School Students | Exclude | Not Stage III Grade C |
|  | Alan 2021 | Effect of Passive Tobacco Smoking Exposure on the Periodontal Status of Turkish Children | Exclude | Not Stage III Grade C |
|  | Alanzi 2018 | Effect of Lactobacillus rhamnosus and Bifidobacterium lactis on gingival health, dental plaque, and periodontopathogens in adolescents: a randomised placebo-controlled clinical trial | Exclude | Study design |
|  | Kovalčíková 2019 | Urea and creatinine levels in saliva of patients with and without periodontitis | Exclude | Not Stage III Grade C |
|  | Alves 2012 | Salivary flow and dental caries in Brazilian youth with type 1 diabetes mellitus: Official Publication of Indian Society for Dental Research | Exclude | Not systemically healthy |
|  | Andronikaki-Faldami 1990 | [Diabetes mellitus and periodontium. 1. Prevalence of periodontal disease in young insulin-dependent diabetics] | Exclude | Not systemically healthy |
|  | Arabsolghar 2016 | Evaluation of salivary immunoglobulin A level in thalassemic patients with periodontitis in comparison with thalassemic patients with healthy periodontium | Exclude | Not Stage III Grade C |
|  | Arai 1996 | Host defensive functions in a family manifesting early-onset periodontitis | Exclude | Age > 25 |
|  | Arana 2017 | Increased salivary oxidative stress parameters in patients with type 2 diabetes: Relation with periodontal disease | Exclude | Not Stage III Grade C |
|  | Aren 2003 | Periodontal health, salivary status, and metabolic control in children with type 1 diabetes mellitus | Exclude | Not Stage III Grade C |
|  | Asok 2016 | A study on comparison of salivary cortisol circadian rhythm in periodontal diseases with external stressors and clinical parameters | Exclude | Not Stage III Grade C |
|  | Aurer 1999 | Inflammatory mediators in saliva of patients with rapidly progressive periodontitis during war stress induced incidence increase | Exclude | Age > 25 |
|  | Azatyan 2019 | Evaluation of IL-2, IL-10, IL-4 and -interferon levels in the oral fluids of patients with hepatitis C, B and HIV | Exclude | Irrelevant title |
|  | Azzopardi 1986 | Gambian children have less salivary secretory immunoglobulin a than British children | Exclude | Not Stage III Grade C |
|  | Baradaran-Rahimi 2010 | Association of interleukin-1 receptor antagonist gene polymorphisms with generalized aggressive periodontitis in an Iranian population | Exclude | Age > 25 |
|  | Barbosa 2020 | Vitamin D receptor FokI and BglI genetic polymorphisms, dental caries, and gingivitis | Exclude | Not Stage III Grade C |
|  | Bastos 2009 | TNF-alpha and IL-4 levels in generalized aggressive periodontitis subjects | Exclude | Unclear age |
|  | Baumer 2011 | Patient-related risk factors for tooth loss in aggressive periodontitis after active periodontal therapy | Exclude | Age > 25 |
|  | Becerik 2012 | Gingival crevicular fluid and plasma acute-phase cytokine levels in different periodontal diseases | Exclude | Age > 25 |
|  | Beliveau 2012 | Benefits of early systemic antibiotics in localized aggressive periodontitis: a retrospective study | Exclude | Not Stage III Grade C |
|  | Borch 2010 | In vitro cytokine responses to periodontal pathogens: generalized aggressive periodontitis is associated with increased IL-6 response to Porphyromonas gingivalis | Exclude | Study design |
|  | Busato 2012 | Impact of clinical status and salivary conditions on xerostomia and oral health-related quality of life of adolescents with type 1 diabetes mellitus | Exclude | Not Stage III Grade C |
|  | CaribÃ© 2020 | Prospective, case-controlled study evaluating serum concentration of sirtuin-1 and mannose-binding lectin in patients with and without periodontal and coronary artery disease | Exclude | Not Stage III Grade C |
|  | Chang 2011 | MUC1 expression in the oral mucosal epithelial cells of the elderly | Exclude | Not Stage III Grade C |
|  | Chinwalla 1998 | Severity of localized juvenile periodontitis as related to polymorphonuclear chemotaxis and specific microbial isolates | Exclude | <10 subjects |
|  | Dakovic 2015 | Gingivitis and periodontitis in children and adolescents suffering from type 1 diabetes mellitus | Exclude | Not Stage III Grade C |
|  | Davies 2011 | Do patients with aggressive periodontitis have evidence of diabetes? A pilot study | Exclude | Age > 25 |
|  | De Angelis 2016 | Salivary and fecal microbiota and metabolome of celiac children under gluten-free diet | Exclude | Not Stage III Grade C |
|  | De La Pena 2007 | Relationship between lactate dehydrogenase activity in saliva and oral health status | Exclude | Not Stage III Grade C |
|  | de Lima Oliveira 2012 | Effects of periodontal therapy on GCF cytokines in generalized aggressive periodontitis subjects | Exclude | Age > 25 |
|  | de Oliveira 2009 | Antimicrobial photodynamic therapy in the non-surgical treatment of aggressive periodontitis: cytokine profile in gingival crevicular fluid, preliminary results | Exclude | Age > 25 |
|  | Delange 2018 | Periodontal disease and its connection to systemic biomarkers of cardiovascular disease in young American Indian/Alaskan natives | Exclude | Age > 25 |
|  | Diehl 2003 | Evidence of a substantial genetic basis for IgG2 levels in families with aggressive periodontitis | Exclude | Genetic profile |
|  | Erciyas 2010 | Association between TNF-alpha, TGF-beta1, IL-10, IL-6 and IFN-gamma gene polymorphisms and generalized aggressive periodontitis | Exclude | Genetic profile |
|  | Erdemir 2010 | Periodontal health in children exposed to passive smoking | Exclude | Not Stage III Grade C |
|  | Ertugrul 2017 | Comparing the efficiency of Er,Cr:YSGG laser and diode laser on human beta-defensin-1 and IL-1beta levels during the treatment of generalized aggressive periodontitis and chronic periodontitis | Exclude | Age > 25 |
|  | Feng 2008 | Prevalence of *Actinobacillus actinomycetemcomitans* in saliva of different types of periodontitis | Exclude | Unclear age |
|  | Guzeldemir 2008 | Interleukin-1 and tumor necrosis factor-alpha gene polymorphisms in Turkish patients with localized aggressive periodontitis | Exclude | Age > 25 |
|  | Hamalaw 2021 | Association of dental and gingival health status with level of salivary characteristics and Streptococcus mutans in children | Exclude | Not Stage III Grade C |
|  | Heikkinen 2016 | Inflammatory mediator polymorphisms associate with initial periodontitis in adolescents | Exclude | Genetic profile |
|  | Iverieli 2009 | [Markers of periodontal diseases and sensitivity to taromentine in patients with aggressive periodontitis] | Exclude | Age > 25 |
|  | Jain 2013 | Association of interleukin-4 and interleukin-17F polymorphisms in periodontitis in Dravidian ethnicity | Exclude | Genetic profile |
|  | Johnson 1980 | Immunopathology of periodontal disease. I. Immunologic profiles in periodontitis and juvenile periodontitis | Include | Met the criteria |
|  | Kamma 2009 | Cytokines in gingival crevicular fluid of adolescents and young adults | Exclude | Not Stage III Grade C |
|  | Kaner 2011 | Calprotectin levels in gingival crevicular fluid predict disease activity in patients treated for generalized aggressive periodontitis | Exclude | Age > 25 |
|  | Khalili 2010 | Salivary calcium: a risk indicator in periodontal disease | Exclude | Not Stage III Grade C |
|  | Khongkhunthian 2013 | Elevated levels of a disintegrin and metalloproteinase 8 in gingival crevicular fluid of patients with periodontal diseases | Exclude | Age > 25 |
|  | 2014 | The novel role of HtrA1 in gingivitis, chronic and aggressive periodontitis | Exclude | Age > 25 |
|  | Nakagawa 1996 | Immunological, genetic, and microbiological study of family members manifesting early-onset periodontitis | Exclude | <10 subjects |
|  | Nibali 2008 | A familial analysis of aggressive periodontitis - clinical and genetic findings | Exclude | Genetic profile |
|  | Obradovic 2021 | The evaluation of saliva oxidative and antioxidative markers' levels in adolescents with gingival inflammation | Exclude | Not Stage III Grade C |
|  | Yücel 2015 | Analysis of TNF--α (-308) polymorphism and gingival crevicular fluid TNF-Î± levels in aggressive and chronic periodontitis: A preliminary report | Exclude | Age > 25 |
|  | Papapanou 2004 | Gene expression signatures in chronic and aggressive periodontitis: a pilot study | Exclude | Age > 25 |
|  | Ranney 1981 | Immunological studies of young adults with severe periodontitis. I. Medical evaluation and humoral factors | Exclude | Age > 25 |
|  | Reichert 2007 | Are there common human leucocyte antigen associations in juvenile idiopathic arthritis and periodontitis? | Exclude | Unclear age |
|  | Sewon 1987 | Calcium concentrations in dental plaque of patients with juvenile and adult periodontitis | Exclude | Age > 25 |
|  | StÃ¤dtler 1984 | Juvenile periodontitis | Exclude | <10 subjects |
|  | Tervahartiala 2000 | The in vivo expression of the collagenolytic matrix metalloproteinases (MMP-2, -8, -13, and -14) and matrilysin (MMP-7) in adult and localized juvenile periodontitis | Exclude | <10 subjects |
|  | Yucel 2020 | Salivary biomarkers in the context of gingival inflammation in children with cystic fibrosis | Exclude | Not Stage III Grade C |
|  | Yue 2013 | Comparative evaluation of cytokines in gingival crevicular fluid and saliva of patients with aggressive periodontitis | Exclude | Age > 25 |
|  | Zafiropoulos 1988 | [Oxidative metabolism of the peripheral neutrophilic granulocytes in patients with rapidly progressing and with localized juvenile periodontitis] | Exclude | "Age > 25 |
|  | Zafiropoulos 1988 | Proteases and inhibitors in the plasma of patients with periodontal disease | Exclude | "Age > 25 |
|  | Zafiropoulos 1988 | Neutrophil chemotaxis function in patients with localized juvenile periodontitis | Exclude | Outcome |
|  | Zhang 1987 | [Research on the functions of neutrophil leukocytes in patients with juvenile periodontitis] | Exclude | Age > 25 |
|  | Zhang 2013 | Elevation of vitamin D-binding protein levels in the plasma of patients with generalized aggressive periodontitis | Exclude | Age > 25 |
|  | Zhou May | [Study of acid alpha-naphthyl-acetate esterase labelled T-lymphocytes and plasma cAMP in juvenile periodontitis] | Exclude | Age > 25 |
|  | Zucchelli 1991 | [Functional PMN defects in young periodontal patients] | Exclude | Outcome |
|  | Acar 2022 | Cytokine profile in serum and gingival crevicular fluid of children with inflammatory bowel disease: A case-control study | Exclude | Not systemically healthy |
|  | Akalin 1993 | Hydroxyproline and total protein levels in gingiva and gingival crevicular fluid in patients with juvenile, rapidly progressive, and adult periodontitis | Exclude | Age > 25 |
|  | Albandar 2001 | Associations between serum antibody levels to periodontal pathogens and early-onset periodontitis | Include | Met the criteria |
|  | Albandar 2002 | Associations of serum concentrations of IgG, IgA, IgM and interleukin-1beta with early-onset periodontitis classification and race | Include | Met the criteria |
|  | Albandar 1998 | Crevicular fluid level of beta-glucuronidase in relation to clinical periodontal parameters and putative periodontal pathogens in early-onset periodontitis | Include | Met the criteria |
|  | Alfant 2008 | Matrix metalloproteinase levels in children with aggressive periodontitis | Include | Met the criteria |
|  | Alves 2016 | Identification of microorganisms in biofluids of individuals with periodontitis and chronic kidney disease using matrix-assisted laser desorption/ionization time-of-flight mass spectrometry: RCM | Exclude | Not Stage III Grade C |
|  | Anand 2016 | Total and Differential Leukocyte Counts in the Peripheral Blood of Patients with Generalised Aggressive Periodontitis | Exclude | Unclear age |
|  | Anil 1993 | Total hemolytic complement (CH50) and its fractions C3 and C4 in the sera of patients with localized juvenile periodontitis | Exclude | ? |
|  | Anil 1990 | Circulating immune complexes in localised juvenile periodontitis | Include | Met the criteria |
|  | Ay 2012 | The gingival crevicular fluid levels of interleukin-11 and interleukin-17 in patients with aggressive periodontitis | Exclude | Age > 25 |
|  | Ayazi 2013 | Analysis of interleukin-1beta gene polymorphism and its association with generalized aggressive periodontitis disease | Exclude | Genetic profile |
|  | Baltacioglu 2014 | Total oxidant status and bone resorption biomarkers in serum and gingival crevicular fluid of patients with periodontitis | Exclude | Age > 25 |
|  | BaltacioÊ‡lu 2014 | Lipid peroxidation levels and total oxidant/antioxidant status in serum and saliva from patients with chronic and aggressive periodontitis. Oxidative stress index: A new biomarker for periodontal disease? | Exclude | Age > 25 |
|  | Bartova 1995 | Local antibodies and cytokine responses in crevicular fluid of patients with juvenile periodontitis | Include | Met the criteria |
|  | Baser 2015 | Plasma and salivary total antioxidant capacity in healthy controls compared with aggressive and chronic periodontitis patients | Exclude | Age > 25 |
|  | Belibasakis 2014 | Soluble triggering receptor expressed on myeloid cells 1 (sTREM-1) in gingival crevicular fluid: association with clinical and microbiologic parameters | Exclude | Age > 25 |
|  | Berdeli 2006 | Association of the IL-1RN2 allele with periodontal diseases | Exclude | Genetic profile |
|  | Berdeli 2006 | Endothelial nitric oxide synthase Glu298Asp gene polymorphism in periodontal diseases | Exclude | Genetic profile |
|  | Berglundh 2002 | The presence of local and circulating autoreactive B cells in patients with advanced periodontitis | Exclude | <10 subjects |
|  | Berglundh 2001 | Some local and systemic immunological features of prepubertal periodontitis | Exclude | <10 subjects |
|  | Bimstein 2004 | Leukocyte esterase and protein levels in saliva, as indicators of gingival and periodontal diseases in children | Exclude | <10 subjects |
|  | Bisson 2012 | Increased gingival crevicular fluid levels of soluble triggering receptor expressed on myeloid cells (sTREM) -1 in severe periodontitis | Exclude | Age > 25 |
|  | Bodur 2003 | Neopterin Profile to Evaluate the Effectiveness of Treatment in Aggressive Periodontitis | Exclude | <10 subjects |
|  | Bolyarova-Konova 2020 | Concentrations of Interleukin-1s in Gingival Crevicular Fluid and Saliva - a Potential Diagnostic Biomarker of Periodontal Diseases | Exclude | Not Stage III Grade C |
|  | Bostanci 2008 | Tumor necrosis factor-alpha-converting enzyme (TACE) levels in periodontal diseases | Exclude | Age > 25 |
|  | Bostanci 2007 | Gingival crevicular fluid levels of RANKL and OPG in periodontal diseases: implications of their relative ratio | Exclude | Age > 25 |
|  | Bostanci 2011 | Effect of periodontal treatment on receptor activator of NF-kappaB ligand and osteoprotegerin levels and relative ratio in gingival crevicular fluid | Exclude | Not Stage III Grade C |
|  | Bostanci 2018 | Targeted Proteomics Guided by Label-free Quantitative Proteome Analysis in Saliva Reveal Transition Signatures from Health to Periodontal Disease | Exclude | Age > 25 |
|  | Bozkurt Dogan 2021 | Emerging roles of Interleukin-34 together with receptor activator of nuclear factor-kB ligand and osteoprotegerin levels in periodontal disease | Exclude | Age > 25 |
|  | Buduneli 2001 | Flow-cytometric analysis of lymphocyte subsets and mCD14 expression in patients with various periodontitis categories | Exclude | Age > 25 |
|  | Cairo 2010 | Markers of systemic inflammation in periodontal patients: chronic versus aggressive periodontitis. An explorative cross-sectional study | Exclude | Age > 25 |
|  | Cao 1991 | [Determination of serum antibody against Bacteroides gingivalis from rapidly progressive periodontitis and juvenile periodontitis patients] | Exclude | Age > 25 |
|  | Carvalho 2010 | FAM5C contributes to aggressive periodontitis | Exclude | Genetic profile |
|  | Celenligil 1998 | Analysis of serum antibody responses to periodontopathogens in early-onset periodontitis patients from different geographical locations | Include | Met the criteria |
|  | Champagne 1998 | Cytoskeletal actin reorganization in neutrophils from patients with localized juvenile periodontitis | Exclude | Age > 25 |
|  | Chen 1991 | Humoral immune responses *to Porphyromonas gingivalis* before and following therapy in rapidly progressive periodontitis patients | Exclude | Unclear age |
|  | Chen 2012 | [Relationship between the preterm low birth weight infant and the periodontal pathogen bacteria in maternal saliva] | Exclude | Irrelevant title |
|  | Cheng 2018 | Comparative analysis of immune cell subsets in peripheral blood from patients with periodontal disease and healthy controls | Exclude | Age > 25 |
|  | Chibebe 2010 | Juveniles versus adults: Differences in PGE2 levels in the gingival crevicular fluid during orthodontic tooth movement | Exclude | Not Stage III Grade C |
|  | Chidzhavadze 2006 | [Diagnostic value of definition of lactate dehydrogenase in mixed saliva in children with periodontitis at diabetes mellitus, type I] | Exclude | Not systemically healthy |
|  | Choi 2018 | Real-time PCR quantification of 9 periodontal pathogens in saliva samples from periodontally healthy Korean young adults | Exclude | Microbiology |
|  | Choi 1996 | Immunoglobulin allotypes and immunoglobulin G subclass responses to *Actinobacillus actinomycetemcomitans* and *Porphyromonas gingivalis* in early-onset periodontitis | Exclude | Unclear age |
|  | Christopher 2021 | Association of CST3 Gene with Its Protein: Cystatin C in Health and Severe Periodontal Disease | Exclude | Not Stage III Grade C |
|  | Cifcibasi 2015 | The role of activated cytotoxic T cells in etiopathogenesis of periodontal disease: does it harm or does it heal? | Exclude | Age > 25 |
|  | Cioloca 2016 | Systemic diabetic context-induced biochemical periodontal alterations in children | Exclude | Not Stage III Grade C |
|  | Cioloca 2015 | Systemic and periodontal inflammatory burden in children and teenagers with diabetes, and clinical correlations | Exclude | Not systemically healthy |
|  | Clark 1977 | Defective neutrophil chemotaxis in juvenile periodontitis | Exclude | Age > 25 |
|  | Cogen 1986 | Host factors in juvenile periodontitis | Exclude | Study design |
|  | Cutando 2006 | Relationship between salivary melatonin and severity of periodontal disease | Exclude | Not Stage III Grade C |
|  | Dabrowski 1977 | IgA, IgG and IgM levels in mixed saliva and oral hygiene, periodontal diseases and dental caries in adolescents | Exclude | Missing full-text |
|  | Dakovic 2013 | Salivary interleukin-8 levels in children suffering from type 1 diabetes mellitus | Exclude | Not systemically healthy |
|  | Danielsen 1993 | Serum immunoglobulin-g antibodies to *porphyromonas-gingivalis, prevotella-intermedia, fusobacterium-nucleatum* and *streptococcus-sanguis* during experimental gingivitis in young-adults | Exclude | Not Stage III Grade C |
|  | Darvishi 2016 | Lack of association between the TNF-alpha-1031genotypes and generalized aggressive periodontitis disease | Exclude | Genetic profile |
|  | Davidopoulou 2012 | Salivary concentration of the antimicrobial peptide LL-37 in children | Exclude | Not Stage III Grade C |
|  | Davidopoulou 2013 | Salivary concentration of free LL-37 in edentulism, chronic periodontitis and healthy periodontium | Exclude | Not Stage III Grade C |
|  | DeNardin 1990 | Antibodies directed to the chemotactic factor receptor detect differences between chemotactically normal and defective neutrophils from LJP patients | Exclude | Genetic profile |
|  | Di Murro 1987 | Rapidly progressive periodontitis. Neutrophil chemotaxis inhibitory factors associated with the presence of Bacteroides gingivalis in crevicular fluid | Exclude | Age > 25 |
|  | Diehl 1999 | Linkage disequilibrium of interleukin-1 genetic polymorphisms with early-onset periodontitis | Exclude | Genetic profile |
|  | Donley 2004 | IgG antibody levels to *Porphyromonas gingivalis* and clinical measures in children | Exclude | Unclear age |
|  | Duarte 2010 | Serum levels of cytokines in subjects with generalized chronic and aggressive periodontitis before and after non-surgical periodontal therapy: a pilot study | Exclude | Age > 25 |
|  | Ebadian 2013 | Gene polymorphisms of TNF-Î± and IL-1Î² are not associated with generalized aggressive periodontitis in an Iranian subpopulation | Exclude | Genetic profile |
|  | Ebersole 1994 | Gingival crevicular fluid antibody to *Actinobacillus actinomycetemcomitans* in periodontal disease | Exclude | Age > 25 |
|  | Ebersole 2015 | Targeted salivary biomarkers for discrimination of periodontal health and disease(s) | Exclude | Not Stage III Grade C |
|  | Ebersole 1991 | Serum antibody in *Actinobacillus actinomycetemcomitans*-infected patients with periodontal disease | Exclude | Age > 25 |
|  | Ebersole 1983 | Human immune responses to oral microorganisms. II. Serum antibody responses to antigens from *Actinobacillus actinomycetemcomitans* and the correlation with localized juvenile periodontitis | Exclude | Age > 25 |
|  | Eger 1996 | Potential diagnostic value of sampling oral mucosal surfaces for *Actinobacillus actinomycetemcomitans* in young adults | Exclude | Not Stage III Grade C |
|  | Eickholz 2013 | Non-surgical periodontal therapy decreases serum elastase levels in aggressive but not in chronic periodontitis | Exclude | Age > 25 |
|  | Emingil 2004 | Gingival crevicular fluid monocyte chemoattractant protein-1 and RANTES levels in patients with generalized aggressive periodontitis | Exclude | Age > 25 |
|  | Emingil 2007 | Toll-like receptor 2 and 4 gene polymorphisms in generalized aggressive periodontitis | Exclude | Genetic profile |
|  | Emingil 2007 | Gene polymorphisms of tissue plasminogen activator and plasminogen activator inhibitor-1 in Turkish patients with generalized aggressive periodontitis | Exclude | Genetic profile |
|  | Emingil 2014 | Matrix metalloproteinase (MMP)-8 and tissue inhibitor of MMP-1 (TIMP-1) gene polymorphisms in generalized aggressive periodontitis: gingival crevicular fluid MMP-8 and TIMP-1 levels and outcome of periodontal therapy | Exclude | Genetic profile |
|  | Emingil 2012 | Effect of azithromycin, as an adjunct to nonsurgical periodontal treatment, on microbiological parameters and gingival crevicular fluid biomarkers in generalized aggressive periodontitis | Exclude | Study design |
|  | Emingil 2006 | Gingival crevicular fluid laminin-5 gamma2-chain levels in periodontal disease | Exclude | Age > 25 |
|  | Emingil 2006 | Gingival crevicular fluid matrix metalloproteinase-25 and -26 levels in periodontal disease | Exclude | Age > 25 |
|  | Emingil 2006 | Gingival crevicular fluid matrix metalloproteinase (MMP)-7, extracellular MMP inducer, and tissue inhibitor of MMP-1 levels in periodontal disease | Exclude | Age > 25 |
|  | Ennibi 2019 | High salivary levels of JP2 genotype of *Aggregatibacter actinomycetemcomitans* is associated with clinical attachment loss in Moroccan adolescents | Exclude | Microbiology |
|  | Enwonwu 2005 | Inflammatory cytokine profile and circulating cortisol levels in malnourished children with necrotizing ulcerative gingivitis | Exclude | Not Stage III Grade C |
|  | Erciyas 2010 | Genetic variation of myeloperoxidase gene contributes to aggressive periodontitis: a preliminary association study in Turkish population | Exclude | Genetic profile |
|  | Farida 1986 | Serological investigation of various forms of inflammatory periodontitis | Exclude | Age > 25 |
|  | Farida 1986 | Serum IgG antibodies to lipopolysaccharides in various forms of periodontal disease in man | Exclude | Age > 25 |
|  | Fiebig 2008 | Polymorphisms in the interleukin-1 (IL1) gene cluster are not associated with aggressive periodontitis in a large Caucasian population | Exclude | Age > 25 |
|  | Fine 2002 | Lactoferrin iron levels are reduced in saliva of patients with localized aggressive periodontitis | Exclude | Age > 25 |
|  | Fine 2013 | Can salivary activity predict periodontal breakdown in *A. actinomycetemcomitans* infected adolescents? | Include | Met the criteria |
|  | Fine 2014 | Macrophage inflammatory protein-1alpha shows predictive value as a risk marker for subjects and sites vulnerable to bone loss in a longitudinal model of aggressive periodontitis | Exclude | Study design |
|  | Fine 2007 | *Aggregatibacter actinomycetemcomitans* and its relationship to initiation of localized aggressive periodontitis: longitudinal cohort study of initially healthy adolescents | Exclude | Microbiology |
|  | Fitzsimmons 2010 | Local and systemic biomarkers in gingival crevicular fluid increase odds of periodontitis | Exclude | Not Stage III Grade C |
|  | Foia 2008 | Analysis of oral expression of the diabetes-periodontal disease binomial relationship in a juvenile population | Exclude | Not Stage III Grade C |
|  | Frias 1994 | No association between secretor status of ABO blood group antigens and juvenile periodontitis | Exclude | Received tx. |
|  | Friedman 1983 | Lysozyme and lactoferrin quantitation in the crevicular fluid | Include | Met the criteria |
|  | Frisken 1987 | Suspected periodontopathic microorganisms and their oral habitats in young children | Exclude | Microbiology |
|  | Fu 1999 | [Relevance of Fc gamma R polymorphism to the susceptibility of early-onset periodontitis] | Exclude | Genetic profile |
|  | Fu 2002 | Fc gamma receptor genes as risk markers for localized aggressive periodontitis in African-Americans | Exclude | Genetic profile |
|  | Fujigaki 2009 | Polymorphism of salivary histatin gene and periodontal disease in the Japanese population | Exclude | Genetic profile |
|  | Gangbar 1990 | Identification of polymorphonuclear leukocyte collagenase and gelatinase activities in mouthrinse samples: correlation with periodontal disease activity in adult and juvenile periodontitis | Exclude | <10 subjects |
|  | Garito 1995 | Salivary PAF levels correlate with the severity of periodontal inflammation | Exclude | Age > 25 |
|  | Gaspirc 2002 | Immunolocalization of inducible nitric oxide synthase in localized juvenile periodontitis patients | Exclude | <10 subjects |
|  | Genco 1980 | Neutrophil chemotaxis impairment in juvenile periodontitis: evaluation of specificity, adherence, deformability, and serum factors | Exclude | Age > 25 |
|  | Geng 2018 | Interleukin-10 polymorphisms affect the key periodontal pathogens in Chinese periodontitis patients | Exclude | Unclear age |
|  | Gmur 1997 | Serum immunoglobulin G responses to various *Actinobacillus actinomycetemcomitans* serotypes in a young ethnographically heterogeneous periodontitis patient group | Exclude | Microbiology |
|  | Goncalves 2013 | Periodontal treatment reduces matrix metalloproteinase levels in localized aggressive periodontitis | Include | Met the criteria |
|  | Gonzales 2010 | The interleukin-4 -34TT and -590TT genotype is correlated with increased expression and protein production in aggressive periodontitis | Exclude | Age > 25 |
|  | Gonzalez 2003 | Comparison of interleukin-1 genotypes in two populations with aggressive periodontitis | Exclude | Genetic profile |
|  | Gooty 2019 | Estimation of phagocytic activity of polymorphonuclear leukocytes in chronic and aggressive periodontitis patients with nitroblue tetrazolium test | Exclude | Study design |
|  | Groenink 1999 | Salivary lactoferrin and low-Mr mucin MG2 in *Actinobacillus actinomycetemcomitans*-associated periodontitis | Exclude | Not Stage III Grade C |
|  | Gu 1998 | Antigenic components of *Actinobacillus actinomycetemcomitans* lipopolysaccharide recognized by sera from patients with localized juvenile periodontitis | Exclude | Unclear age |
|  | Gunsolley 1987 | Relationship of serum antibody to attachment level patterns in young adults with juvenile periodontitis or generalized severe periodontitis | Exclude | Age > 25 |
|  | Gunsolley 1990 | Serum antibodies to periodontal bacteria | Exclude | Age > 25 |
|  | Guo 2000 | Antibody responses against *Porphyromonas gingivalis* infection in patients with early-onset periodontitis | Exclude | Age > 25 |
|  | Gupta 2015 | Effect of surgical periodontal therapy on serum C-reactive protein levels using ELISA in both chronic and aggressive periodontitis patient | Exclude | Age > 25 |
|  | Gurkan 2009 | Angiotensin-converting enzyme (ACE), angiotensinogen (AGT), and angiotensin II type 1 receptor (AT1R) gene polymorphisms in generalized aggressive periodontitis | Exclude | Genetic profile |
|  | Gutierrez 1991 | Phagocytosis and adhesiveness of peripheral blood polymorphonuclear leukocytes in patients with rapidly progressive periodontitis | Exclude | Study design |
|  | Haba 2011 | Evaluation of serum and gingival crevicular fluid C-reactive protein and IL-6 levels in patients with periodontitis and transient ischemic attacks | Exclude | Not Stage III Grade C |
|  | Hagewald 2000 | Total IgA and Porphyromonas gingivalis-reactive IgA in the saliva of patients with generalised early-onset periodontitis | Exclude | Age > 25 |
|  | Hagewald 2003 | Salivary IgA in response to periodontal treatment | Exclude | Age > 25 |
|  | Harris 2020 | Association of P2RX7 functional variants with localized aggressive periodontitis | Exclude | Genetic profile |
|  | Havemose-Poulsen 2005 | Cytokine profiles in peripheral blood and whole blood cell cultures associated with aggressive periodontitis, juvenile idiopathic arthritis, and rheumatoid arthritis | Exclude | Age > 25 |
|  | Hayashi 1999 | Increased levels of soluble CD14 in sera of periodontitis patients | Exclude | Age > 25 |
|  | Heikkinen 2019 | Cross-sectional analysis of risk factors for subclinical periodontitis; active matrix metalloproteinase-8 as a potential indicator in initial periodontitis in adolescents | Exclude | Not Stage III Grade C |
|  | 2017 | Pilot Study on the Genetic Background of an Active Matrix Metalloproteinase-8 Test in Finnish Adolescents | Exclude | Genetic profile |
|  | Heikkinen 2010 | Smoking affects diagnostic salivary periodontal disease biomarker levels in adolescents | Exclude | Not Stage III Grade C |
|  | Hendek 2015 | Evaluation of salivary procalcitonin levels in different periodontal diseases | Exclude | Age > 25 |
|  | Hendek 2019 | Salivary LL-37 and periodontal health in children exposed to passive smoking | Exclude | Not Stage III Grade C |
|  | Hendler 2010 | Involvement of autoimmunity in the pathogenesis of aggressive periodontitis | Exclude | Age > 25 |
|  | Henry 1984 | ABO BLOOD GROUPING OF YOUNG-ADULT PERIODONTITIS PATIENTS | Exclude | Missing full-text |
|  | Henskens 1993 | Protein, albumin and cystatin concentrations in saliva of healthy subjects and of patients with gingivitis or periodontitis | Exclude | Not Stage III Grade C |
|  | Hodge 2001 | Failure to detect an association with IL1 genotypes in European Caucasians with generalised early onset periodontitis | Exclude | Genetic profile |
|  | Hooshm 2008 | Interleukin-4 (C-590T) and interferon-Î³ (G5644A) gene polymorphisms in patients with periodontitis | Exclude | Genetic profile |
|  | Houshm 2012 | Influence of cytotoxic T lymphocyte antigen-4 (CTLA-4) gene polymorphisms in periodontitis | Exclude | Genetic profile |
|  | Houshm 2009 | E-selectin and L-selectin polymorphisms in patients with periodontitis | Exclude | Genetic profile |
|  | Huang 2020 | Analysis of salivary exosomal proteins in young adults with severe periodontitis | Exclude | Age > 25 |
|  | Ilgenli 2006 | Gingival crevicular fluid matrix metalloproteinase-13 levels and molecular forms in various types of periodontal diseases | Exclude | Age > 25 |
|  | Ingman 1996 | Matrix metalloproteinases and their inhibitors in gingival crevicular fluid and saliva of periodontitis patients | Exclude | Age > 25 |
|  | Isaza-Guzman 2008 | Estimation of sCD14 levels in saliva obtained from patients with various periodontal conditions | Exclude | Age > 25 |
|  | Isaza-Guzman 2017 | Salivary Levels of NLRP3 Inflammasome-Related Proteins as Potential Biomarkers of Periodontal Clinical Status | Exclude | Age > 25 |
|  | Ishikawa 1988 | Diversity of IgG antibody responses in the patients with various types of periodontitis | Exclude | <10 subjects |
|  | Iwata 2009 | Ceruloplasmin induces polymorphonuclear leukocyte priming in localized aggressive periodontitis | Exclude | Age > 25 |
|  | Jakonis 1983 | The quantitative composition of immunoglobulins in the peripheral venous and capillary blood serum in the gingiva, and in the mixed saliva of gingivitis and periodontitis patients | Exclude | Age > 25 |
|  | Jaradat 2012 | Association of interleukin-10 gene promoter polymorphisms with chronic and aggressive periodontitis | Exclude | Genetic profile |
|  | Jentsch 2015 | Reduced numbers of peripheral blood CD27+ IgD- memory B cells in patients with aggressive periodontitis | Exclude | Age > 25 |
|  | Kalash 2015 | Influence of Periodontal Therapy on Systemic Lipopolysaccharides in Children with Localized Aggressive Periodontitis | Include | Met the criteria |
|  | Kalburgi 2013 | Expression profile of IL-35 mRNA in gingiva of chronic periodontitis and aggressive periodontitis patients: a semiquantitative RT-PCR study | Exclude | Genetic profile |
|  | Karasneh 2011 | Investigation of the interleukin-1 gene cluster polymorphisms in Jordanian patients with chronic and aggressive periodontitis | Exclude | Genetic profile |
|  | Kaslick 1980 | Association between ABO blood groups, HL-A antigens and periodontal diseases in young adults: a follow-up study | Exclude | Age > 25 |
|  | Kazemi 2015 | Association between Manganese Superoxide Dismutase (MnSOD Val-9Ala) genotypes with the risk of generalized aggressive periodontitis disease | Exclude | Genetic profile |
|  | Kilian 1989 | Distribution of immunoglobulin isotypes including IgA subclasses in adult, juvenile, and rapidly progressive periodontitis | Exclude | <10 subjects |
|  | Kim 2021 | Identification of galectin-10 as a biomarker for periodontitis based on proteomic analysis of gingival crevicular fluid | Exclude | Age > 25 |
|  | Kjeldsen 1995 | Bacterial-stimulated cytokine production of peripheral mononuclear cells from patients of various periodontitis categories | Exclude | <10 subjects |
|  | Kolesnyk JAN | Changes in the expression of mRNA TLR2 and 4 type, nuclear factor kB and pro-inflammatory cytokines IL-1 beta and IL-17A in the epithelium of the oral cavity in children with special needs | Exclude | Not Stage III Grade C |
|  | Konopka 2007 | Total antioxidant status and 8-hydroxy-2'-deoxyguanosine levels in gingival and peripheral blood of periodontitis patients | Exclude | Not Stage III Grade C |
|  | Koutouzis 2009 | Autoreactivity of serum immunoglobulin to periodontal tissue components: a pilot study | Exclude | <10 subjects |
|  | Kumar 2016 | Evaluation of chemokines in gingival crevicular fluid in children with band and loop space maintainers: A clinico-biochemical study | Exclude | Not Stage III Grade C |
|  | Kuru 1999 | Microbiological features and crevicular fluid aspartate aminotransferase enzyme activity in early onset periodontitis patients | Exclude | Age > 25 |
|  | Kuula 2008 | Human beta-defensin-1 and -2 and matrix metalloproteinase-25 and -26 expression in chronic and aggressive periodontitis and in peri-implantitis | Exclude | Genetic profile |
|  | Kwon 2017 | Elevated leukocyte count is associated with periodontitis in Korean adults: the 2012-2014 KNHANES | Exclude | Not Stage III Grade C |
|  | LarivÃ©e 1986 | Collagenase and collagenase inhibitor activities in crevicular fluid of patients receiving treatment for localized juvenile periodontitis | Exclude | <10 subjects |
|  | Layik 2000 | Analysis of human gingival tissue and gingival crevicular fluid beta-glucuronidase activity in specific periodontal diseases | Exclude | Age > 25 |
|  | Lee 2020 | Diagnostic Models for Screening of Periodontitis with Inflammatory Mediators and Microbial Profiles in Saliva | Exclude | Age > 25 |
|  | Lee 2020 | Differential expression of microRNAs in the saliva of patients with aggressive periodontitis: a pilot study of potential biomarkers for aggressive periodontitis | Exclude | Genetic profile |
|  | Lehner 1974 | Immunological aspects of juvenile periodontitis (periodontosis) | Include | Met the criteria |
|  | Li 2004 | Association analysis between interleukin-1 family polymorphisms and generalized aggressive periodontitis in a Chinese population | Exclude | Genetic profile |
|  | Liu 2010 | Initial periodontal therapy reduced systemic and local 25-hydroxy vitamin D(3) and interleukin-1beta in patients with aggressive periodontitis | Exclude | Age > 25 |
|  | Liu 2009 | [Correlation analysis between plasma levels of 25-hydroxy vitamin D3 and osteocalcin in patients with aggressive periodontitis] | Exclude | Age > 25 |
|  | Liu 1999 | [The elastase activity in gingival crevicular fluid from rapidly progressive periodontitis] | Exclude | Age > 25 |
|  | Liu 2013 | [Association between plasma leptin level and periodontal parameters in patients with aggressive periodontitis] | Exclude | Age > 25 |
|  | Lopez 1992 | Clinical, laboratory, and immunological studies of a family with a high prevalence of generalized prepubertal and juvenile periodontitis | Exclude | <10 subjects |
|  | Lopez 2011 | Serum levels of C-reactive protein in adolescents with periodontitis | Exclude | Smokers |
|  | Lourenco 2013 | Lactoferrin, a marker for periodontal disease | Exclude | Age > 25 |
|  | Lu 1993 | IMMUNOGLOBULIN CLASS AND SUBCLASS DISTRIBUTION OF ANTIBODIES REACTIVE WITH THE IMMUNODOMINANT ANTIGEN OF ACTINOBACILLUS-ACTINOMYCETEMCOMITANS SEROTYPE-B | Exclude | Age > 25 |
|  | Lu 1994 | SERUM IMMUNOGLOBULIN-G SUBCLASS CONCENTRATIONS IN PERIODONTALLY HEALTHY AND DISEASED INDIVIDUALS | Exclude | Age > 25 |
|  | Lu 2014 | Effect of non-surgical periodontal treatment on short chain fatty acid levels in gingival crevicular fluid of patients with generalized aggressive periodontitis | Exclude | Age > 25 |
|  | Lu 2013 | Relationship between volatile fatty acids and *Porphyromonas gingivalis* and Treponema denticola in gingival crevicular fluids of patients with aggressive periodontitis | Exclude | Age > 25 |
|  | Luo 2011 | Expression of HMGB1 and HMGN2 in gingival tissues, GCF and PICF of periodontitis patients and peri-implantitis | Exclude | Genetic profile |
|  | Machtei 2008 | The use of 2 antibiotic regimens in aggressive periodontitis: comparison of changes in clinical parameters and gingival crevicular fluid biomarkers | Exclude | Age > 25 |
|  | Majumder 2017 | Alliance of matrix metalloproteinase-9 (MMP-9) promoter gene polymorphism with chronic and aggressive periodontitis in Indian population | Exclude | Genetic profile |
|  | Majumder 2018 | Association of tumor necrosis factor-alpha (TNF-alpha) gene promoter polymorphisms with aggressive and chronic periodontitis in the eastern Indian population | Exclude | Genetic profile |
|  | Maksimovskaia 1998 | The relationship of the clinical status of the periodontium and the cytochemical indices of the enzyme activity of the peripheral blood leukocytes | Exclude | Not Stage III Grade C |
|  | Manson 1977 | Juvenile periodontitis (periodontosis) | Exclude | No analysis |
|  | Maria de Freitas 2007 | Analysis of IL-1A(-889) and TNFA(-308) gene polymorphism in Brazilian patients with generalized aggressive periodontitis | Exclude | Genetic profile |
|  | Martins 2019 | One-year follow-up of the immune profile in serum and selected sites of generalized and localized aggressive periodontitis | Exclude | Age > 25 |
|  | Mason 1984 | Local Immunoglobulin Synthesis in Juvenile Periodontitis: Initial Findings | Exclude | <10 subjects |
|  | McFarlane 1991 | Interleukin-2, interleukin-2 receptor and interleukin-4 levels are elevated in the sera of patients with periodontal disease | Exclude | Age > 25 |
|  | McFarlane 1990 | The release of interleukin-1 beta, tumor necrosis factor-alpha and interferon-gamma by cultured peripheral blood mononuclear cells from patients with periodontitis | Exclude | Age > 25 |
|  | Mellati 2007 | Analysis of -1082 IL-10 gene polymorphism in Iranian patients with generalized aggressive periodontitis | Exclude | Genetic profile |
|  | Miranda 2006 | Changes in periodontal and rheumatological conditions after 2 years in patients with juvenile idiopathic arthritis | Exclude | Not systemically healthy |
|  | Miranda 2005 | Increased interleukin-18 in patients with juvenile idiopathic arthritis and early attachment loss | Exclude | Not systemically healthy |
|  | Mitsuhata 2014 | Effectiveness of aspartate aminotransferase as a marker of periodontal disease in children and adolescents | Exclude | Not Stage III Grade C |
|  | Mizuno 2011 | Proteome analysis of proteins related to aggressive periodontitis combined with neutrophil chemotaxis dysfunction | Exclude | study design |
|  | Mooney 2001 | Humoral immune response in early-onset periodontitis: influence of smoking | Exclude | Age > 25 |
|  | Moreira 2015 | Antimicrobial photodynamic therapy as an adjunct to non-surgical treatment of aggressive periodontitis: a split-mouth randomized controlled trial | Exclude | Genetic profile |
|  | Nibali 2008 | Association between interleukin-6 promoter haplotypes and aggressive periodontitis | Exclude | Age > 25 |
|  | Nisengard 1980 | Humoral immunologic responses in idiopathic juvenile periodontitis (periodontosis) | Exclude | Age > 25 |
|  | Offenbacher 1986 | The use of crevicular fluid prostaglandin E2 levels as a predictor of periodontal attachment loss | Exclude | unclear age |
|  | Okui 2012 | The Presence of IL-17(+)/FOXP3(+) Double-positive Cells in Periodontitis | Exclude | Not Stage III Grade C |
|  | Over 1993 | Myeloperoxidase activity in peripheral blood, neutrophil crevicular fluid and whole saliva of patients with periodontal disease | Exclude | Age > 25 |
|  | Ozturk 2015 | The actin-bundling protein L-plastin: a novel local inflammatory marker associated with periodontitis | Exclude | Age > 25 |
|  | Page 1978 | Effects of prostaglandin on the antigen- and mitogen-driven responses of peripheral blood lymphocytes from patients with adult and juvenile periodontitis | Exclude | Age > 25 |
|  | Page 1985 | Clinical and laboratory studies of a family with a high prevalence of juvenile periodontitis | Exclude | Age > 25 |
|  | Palmer 1990 | Expression of complement receptors CR1 and CR3 in patients with juvenile periodontitis | Exclude | Age > 25 |
|  | Park 2017 | Association of Some Vitamins and Minerals with Periodontitis in a Nationally Representative Sample of Korean Young Adults | Exclude | Not Stage III Grade C |
|  | Parkhill 2000 | Association of interleukin-1 gene polymorphisms with early-onset periodontitis | Exclude | Age > 25 |
|  | Perozini 2010 | Gingival crevicular fluid biochemical markers in periodontal disease: a cross-sectional study | Exclude | Not Stage III Grade C |
|  | Pietruska 2005 | Expressions of selected adhesion molecules on peripheral blood leukocytes in patients with aggressive periodontitis | Exclude | Genetic profile |
|  | Pontes 2004 | Interleukin-4 gene polymorphism and its relation to periodontal disease in a Brazilian population of African heritage | Exclude | Genetic profile |
|  | Prodan 2016 | A Study of the Variation in the Salivary Peptide Profiles of Young Healthy Adults Acquired Using MALDI-TOF MS | Exclude | Age > 25 |
|  | Qi 2021 | 1Î±,25-dihydroxyvitamin D3 promotes early osteogenic differentiation of PDLSCs and a 12-year follow-up case of early-onset vitamin D deficiency periodontitis | Exclude | study design |
|  | Quappe 2004 | Association of interleukin-1 polymorphisms with aggressive periodontitis | Exclude | Genetic profile |
|  | Quinn 1996 | Influence of smoking and race on immunoglobulin G subclass concentrations in early-onset periodontitis patients | Exclude | Age > 25 |
|  | Rai 2008 | Biomarkers of periodontitis in oral fluids | Exclude | Not Stage III Grade C |
|  | Raisanen 2020 | A point-of-care test of active matrix metalloproteinase-8 predicts triggering receptor expressed on myeloid cells-1 levels in saliva | Exclude | Smokers |
|  | RÃ¤isÃ¤nen 2019 | Active Matrix Metalloproteinase-8 Point-of-Care (PoC)/Chairside Mouthrinse Test vs. Bleeding on Probing in Diagnosing Subclinical Periodontitis in Adolescents | Exclude | Irrelevant samples |
|  | Raivisto 2020 | Regulation of Salivary Peptidoglycan Recognition Protein 1 in Adolescents | Exclude | Not Stage III Grade C |
|  | Raivisto 2020 | Active Matrix Metalloproteinase-8 Chair Side Mouth Rinse Test, Health Behaviour and Oral Health in Finnish Adolescent Cohort | Exclude | Not Stage III Grade C |
|  | Rebeis 2019 | Effect of periodontal treatment on *Aggregatibacter actinomycetemcomitans* colonization and serum IgG levels against *A. actinomycetemcomitans* serotypes and Omp29 of aggressive periodontitis patients | Exclude | Age > 25 |
|  | Ren 2008 | Interleukin-1 family polymorphisms in aggressive periodontitis patients and their relatives | Exclude | Age > 25 |
|  | Rennie 2006 | Pestivirus as a cause of white matter damage - down but not out | Exclude | study design |
|  | Ribeiro 2021 | Systemic circulating inflammatory burden and periodontitis in adolescents | Exclude | Smokers |
|  | Richter 2022 | Exome Sequencing of 5 Families with Severe Early-Onset Periodontitis | Exclude | Genetic profile |
|  | Riis 2015 | Salivary Cytokines as a Minimally-Invasive Measure of Immune Functioning in Young Children: Correlates of Individual Differences and Sensitivity to Laboratory Stress | Exclude | Not Stage III Grade C |
|  | Romano 2018 | Biomarker levels in gingival crevicular fluid of generalized aggressive periodontitis patients after non-surgical periodontal treatment | Exclude | Age > 25 |
|  | Roshna 1954 | A case-control study on the association of human leukocyte antigen-A*9 and -B*15 alleles with generalized aggressive periodontitis in an Indian population | Exclude | Genetic profile |
|  | Rylev 2011 | Microbiological and immunological characteristics of young Moroccan patients with aggressive periodontitis with and without detectable Aggregatibacter actinomycetemcomitans JP2 infection | Exclude | <10 subjects |
|  | Salman 2021 | Salivary Oxidative Stress Markers' Relation to Oral Diseases in Children and Adolescents | Exclude | Not Stage III Grade C |
|  | Sandholm 1983 | Concentrations of serum protease inhibitors and immunoglobulins in juvenile periodontitis | Include | Met the criteria |
|  | Sandholm 1984 | Local immunoglobulin synthesis in juvenile and adult periodontitis | Exclude | Age > 25 |
|  | Sandholm 1986 | Serum antibody levels to 4 periodontal pathogens remain unaltered after mechanical therapy of juvenile periodontitis | Exclude | Age > 25 |
|  | Sandholm 1987 | Salivary IgG, a parameter of periodontal disease activity? High responders to *Actinobacillus actinomycetemcomitans* Y4 in juvenile and adult periodontitis | Exclude | Age > 25 |
|  | Saraiva 2013 | Evaluation of IL17A expression and of IL17A, IL17F and IL23R gene polymorphisms in Brazilian individuals with periodontitis | Exclude | Genetic profile |
|  | Scapoli 2010 | IL-1 Gene cluster is not Linked to Aggressive Periodontitis | Exclude | Genetic profile |
|  | Schmidt 2014 | General immune status and oral microbiology in patients with different forms of periodontitis and healthy control subjects | Exclude | Age > 25 |
|  | Schmidt 2019 | Stress-related hormones in association with periodontal condition in adolescents-results of the epidemiologic LIFE Child study | Exclude | Not Stage III Grade C |
|  | Sewon 1990 | A study of the possible correlation of high salivary calcium levels with periodontal and dental conditions in young adults | Exclude | Not Stage III Grade C |
|  | Shaddox 2010 | Hyper-responsive phenotype in localized aggressive periodontitis | Exclude | study design |
|  | Shaddox 2013 | LPS-induced inflammatory response after therapy of aggressive periodontitis | Exclude | study design |
|  | Shaddox 2016 | Localized aggressive periodontitis immune response to healthy and diseased subgingival plaque | Exclude | study design |
|  | Shapira 1997 | Possible autosomal-dominant inheritance of prepubertal periodontitis in an extended kindred | Exclude | <10 subjects |
|  | Shapira 1996 | Prostaglandin E2 secretion, cell maturation, and CD14 expression by monocyte-derived macrophages from localized juvenile periodontitis patients | Exclude | <10 subjects |
|  | Shapira 2001 | Genetic polymorphism of the tumor necrosis factor (TNF)-alpha promoter region in families with localized early-onset periodontitis | Exclude | Genetic profile |
|  | Shete 2010 | Association of single nucleotide gene polymorphism at interleukin-1beta +3954, -511, and -31 in chronic periodontitis and aggressive periodontitis in Dravidian ethnicity | Exclude | Genetic profile |
|  | Shi 2015 | Association between plasma leptin level and systemic inflammatory markers in patients with aggressive periodontitis | Exclude | Age > 25 |
|  | Shi 2008 | Systemic inflammation markers in patients with aggressive periodontitis: a pilot study | Exclude | Age > 25 |
|  | Shibata 2000 | Defective calcium influx factor activity in neutrophils from patients with localized juvenile periodontitis | Exclude | study design |
|  | Shih 2014 | Association of CCL5 and CCR5 gene polymorphisms with periodontitis in Taiwanese | Exclude | Genetic profile |
|  | Shimomura-Kuroki 2009 | Tannerella forsythia and the HLA-DQB1 allele are associated with susceptibility to periodontal disease in Japanese adolescents | Exclude | Genetic profile |
|  | Shivaprasad 2013 | Effect of non-surgical periodontal therapy on interleukin-29 levels in gingival crevicular fluid of chronic periodontitis and aggressive periodontitis patients | Exclude | Age > 25 |
|  | Silveira 2016 | Analysis of polymorphisms in Interleukin 10, NOS2A, and ESR2 genes in chronic and aggressive periodontitis | Exclude | Genetic profile |
|  | Sims 1991 | Antigens of *Actinobacillus actinomycetemcomitans* recognized by patients with juvenile periodontitis and periodontally normal subjects | Exclude | Age > 25 |
|  | Sjodin 1995 | Periodontal and systemic findings in children with marginal bone loss in the primary dentition | Include | Met the criteria |
|  | Slobodina 2009 | Blood biochemical parameters of C-reactive protein and C-terminal telopeptides in adolescents and young people for early diagnosis of inflammatory diseases of the periodontium | Exclude | Not Stage III Grade C |
|  | Song 2015 | Genes related to inflammation and bone loss process in periodontitis suggested by bioinformatics methods | Exclude | Genetic profile |
|  | Spindler 1986 | Juvenile periodontitis. I. Demonstration of local immunoglobulin synthesis | Include | Met the criteria |
|  | Spinei 2013 | Crystallogenesis of oral fluid in the diagnosis of dental caries and inflammatory periodontal diseases in children | Exclude | Not Stage III Grade C |
|  | Sridharan 2017 | Salivary Alkaline Phosphatase as a Noninvasive Marker for Periodontal Disease in Children with Uncontrolled Type 1 Diabetes Mellitus | Exclude | not systemically healthy |
|  | Stashenko 2011 | Inflammation and genetic risk indicators for early periodontitis in adults | Exclude | Not Stage III Grade C |
|  | Stolf 2021 | Influence of rs6667202 SNP on Interleukin-10 Levels in the Gingival Fluid of Patients with Periodontitis Grade C | Exclude | Genetic profile |
|  | Sun 2009 | Elevation of C-reactive protein and interleukin-6 in plasma of patients with aggressive periodontitis | Exclude | Age > 25 |
|  | Sun 2020 | Alteration of salivary microbiome in periodontitis with or without type-2 diabetes mellitus and metformin treatment | Exclude | Microbiology |
|  | Suomalainen 1996 | Peroxidases, lactoferrin and lysozyme in peripheral blood neutrophils, gingival crevicular fluid and whole saliva of patients with localized juvenile periodontitis | Exclude | <10 subjects |
|  | Taiete 2019 | Local IL-10 level as a predictive factor in generalized aggressive periodontitis treatment response | Exclude | Age > 25 |
|  | Takahashi 1995 | Studies on the phenotypic and functional characterization of peripheral blood lymphocytes from patients with early-onset periodontitis | Exclude | Age > 25 |
|  | Tew 1985 | Relationship between gingival crevicular fluid and serum antibody titers in young adults with generalized and localized periodontitis | Exclude | Age > 25 |
|  | Tew 1984 | SERUM ANTIBODY AGAINST TREPONEMES IN ADOLESCENTS AND YOUNG-ADULTS WITH PERIODONTITIS | Exclude | Missing full-text |
|  | Tew 1996 | Antibody of the IgG2 subclass, Actinobacillus actinomycetemcomitans, and early-onset periodontitis | Exclude | study design |
|  | Tobon-Arroyave 2008 | Correlation between salivary IL-1beta levels and periodontal clinical status | Exclude | Age > 25 |
|  | Tothova 2013 | Salivary markers of oxidative stress and their relation to periodontal and dental status in children | Exclude | Not Stage III Grade C |
|  | Tsai 1981 | Serum neutralizing activity against *Actinobacillus actinomycetemcomitans* leukotoxin in juvenile periodontitis | Exclude | Age > 25 |
|  | Tsai 2021 | Associations between metabolic biomarkers and localized stage II/III periodontitis in young adults: The CHIEF Oral Health study | Exclude | Age > 25 |
|  | Tsybikov 2014 | [Serum, oral and gingival fluid levels of heat shock protein-70, cytokines and their autoantibodies by periodontal disease] | Exclude | Not Stage III Grade C |
|  | Tyagi 1992 | Altered diacylglycerol level and metabolism in neutrophils from patients with localized juvenile periodontitis | Exclude | study design |
|  | Unsal 1996 | The effects of periodontal therapy on serum antibody levels to *Actinobacillus actinomycetemcomitans* and *Porphyromonas gingivalis* (part II) | Exclude | Duplication |
|  | Unsal 1996 | Serum antibodies to *Actinobacillus actinomycetemcomitans* and *Porphyromonas gingivalis* in juvenile periodontitis and adult periodontitis (part I) | Include | Met the criteria |
|  | Ustaoglu 2020 | Does periodontitis affect mean platelet volume(MPV) and plateletcrit (PCT) levels in healthy adults? | Exclude | Irrelevant title |
|  | Van Dyke 1981 | Reduced chemotactic peptide binding in juvenile periodontitis: A model for neutrophil function | Exclude | Age > 25 |
|  | Van Dyke 1987 | Association of an abnormality of neutrophil chemotaxis in human periodontal disease with a cell surface protein | Exclude | study design |
|  | Vincent 1985 | Reaction of human sera from juvenile periodontitis, rapidly progressive periodontitis, and adult periodontitis patients with selected periodontopathogens | Exclude | Age > 25 |
|  | von Wowern 2001 | Bone mineral content and bone metabolism in young adults with severe periodontitis | Exclude | Age > 25 |
|  | Wang 2005 | Elevated serum IgG titer and avidity to *Actinobacillus actinomycetemcomitans* serotype c in Japanese periodontitis patients | Exclude | Microbiology |
|  | Wang 2008 | [Screening and analysis of multi-alleles in generalized aggressive periodontitis] | Exclude | Genetic profile |
|  | Wang 2013 | [The salivary factors related to caries and periodontal disease in children and adolescents with diabetes mellitus] | Exclude | Not Stage III Grade C |
|  | Wang 2013 | [Long-term clinical and hematologic effects of non-surgical treatment on aggressive periodontitis] | Exclude | Age > 25 |
|  | Wendl 2021 | Association between metabolic and hormonal profile, proinflammatory cytokines in saliva and gingival health in adolescent females with polycystic ovary syndrome | Exclude | Irrelevant title |
|  | Westerlund 1996 | Human neutrophil gelatinase and associated lipocalin in adult and localized juvenile periodontitis | Exclude | study design |
|  | Williams 1985 | Assessment of serum antibody patterns and analysis of subgingival microflora of members of a family with a high prevalence of early-onset periodontitis | Exclude | Microbiology |
|  | Wilton 1991 | Serum IgG antibodies reactive with potential periodontal pathogens and other subgingival plaque bacteria in a population of Male british adolescents with minimal destructive periodontitis | Exclude | Not Stage III Grade C |
|  | Wu 2018 | Salivary biomarker combination prediction model for the diagnosis of periodontitis in a Taiwanese population | Exclude | Not Stage III Grade C |
|  | Xu 2009 | Serum anti-Pg IgG antibody titers in patients with aggressive periodontitis | Exclude | Age > 25 |
|  | Xynogala 2009 | Evaluation of the humoral immune response to the cytolethal distending toxin of *Aggregatibacter actinomycetemcomitans* Y4 in subjects with localized aggressive periodontitis | Exclude | Age > 25 |
|  | Yamazaki 2003 | Single-nucleotide polymorphism in the CD14 promoter and periodontal disease expression in a Japanese population | Exclude | Genetic profile |
|  | Yasuda 2003 | FcgammaRIIB gene polymorphisms in Japanese periodontitis patients | Exclude | Not Stage III Grade C |
|  | YlÃ¶stalo 2008 | Self-reported gingivitis and tooth loss poorly predict C-reactive protein levels: A study among Finnish young adults | Exclude | Not Stage III Grade C |
|  | Yuan 2018 | Matrix metalloproteinase-8 levels in oral samples as a biomarker for periodontitis in the Chinese population: an observational study | Exclude | Not Stage III Grade C |
|  | Yucel 2013 | Association of interleukin-1 beta (+3954) gene polymorphism and gingival crevicular fluid levels in patients with aggressive and chronic periodontitis | Exclude | Genetic profile |
|  | Zafiropoulos 1987 | Determination of the ELP (elastase-like proteinase) plasma levels in patients with rapidly advancing and with juvenile periodontitis | Include | Met the criteria |
|  | Zalewska 2020 | Dysfunction of Salivary Glands, Disturbances in Salivary Antioxidants and Increased Oxidative Damage in Saliva of Overweight and Obese Adolescents | Exclude | Not systemically healthy |
|  | Zeigler 2015 | Pathological periodontal pockets are associated with raised diastolic blood pressure in obese adolescents | Exclude | Irrelevant title |
|  | Zhan 2016 | Platelet activation and platelet-leukocyte interaction in generalized aggressive periodontitis | Exclude | Age > 25 |
|  | Zhang 1996 | Hyper-immunoglobulin G2 production by B cells from patients with localized juvenile periodontitis and its regulation by monocytes | Exclude | Age > 25 |
|  | Zorina 2013 | Correlation of gene polymorphism and risk of aggressive periodontal disease | Exclude | Genetic profile |
|  | Mishra 2022 | Systemic immune-inflammation index in patients with generalized stage III grade C periodontitis | Exclude | Age > 25 |
|  | Nimmala 2022 | Lactate Dehydrogenase: A Pragmatic Diagnostic Tool for Periodontitis and Diabetes | Exclude | Not Stage III Grade C |
|  | Kim 2022 | Activation and increased production of interleukin‐17 and tumour necrosis factor‐α of mucosal‐associated invariant T cells in patients with periodontitis | Exclude | Age > 25 |
|  | Tavakoli 2022 | Gender differences in immunological response of African American juveniles with Grade C molar incisor pattern periodontitis | Include | Met the criteria |
|  | Wu 2023 | Saliva microbiota and metabolite in individuals with caries or periodontitis | Exclude | Age > 25 |
|  | Zhang 2022 | Resveratrol decreases local inflammatory markers and systemic endotoxin in patients with aggressive periodontitis | Exclude | Age > 25 |
