# Supplementary figures and images for: Biomarkers for diagnosis of stage III, grade C with molar incisor pattern periodontitis in children and young adults: a systematic review and meta-analysis
Source: Clin Oral Investig. 2023 Aug 3;27(9):4929–55. doi: 10.1007/s00784-023-05169-x (PMC10492694; doi:10.1007/s00784-023-05169-x)

**Appendix 4. Twelve meta-analysis models were performed for biomarkers in GCF samples.**


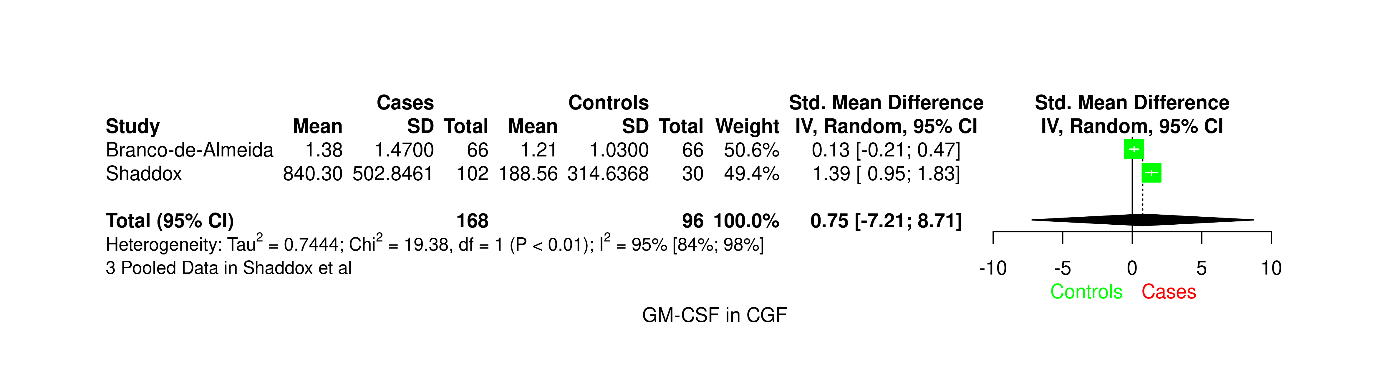

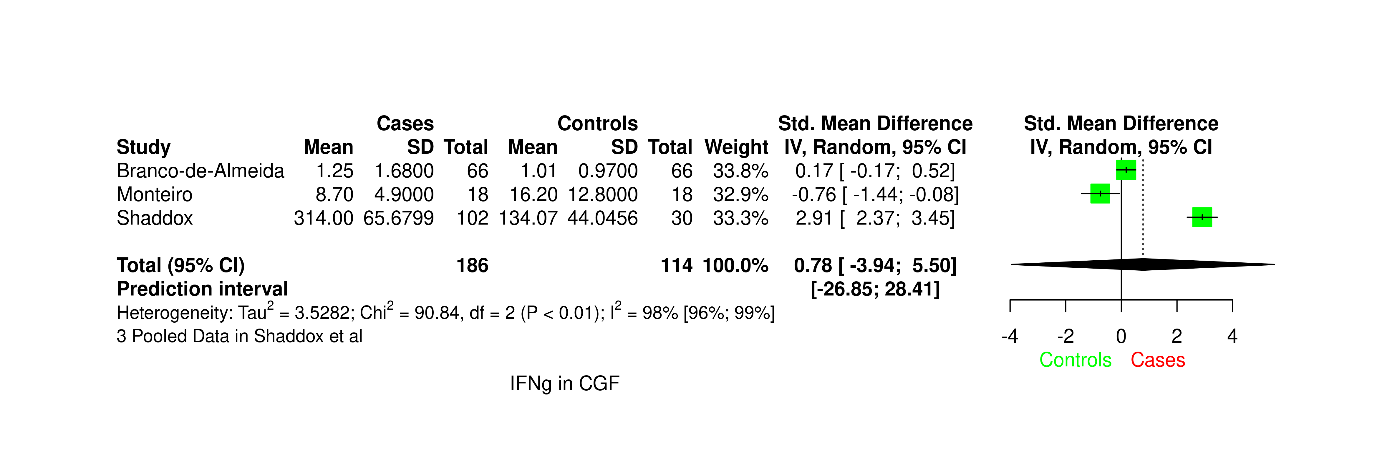

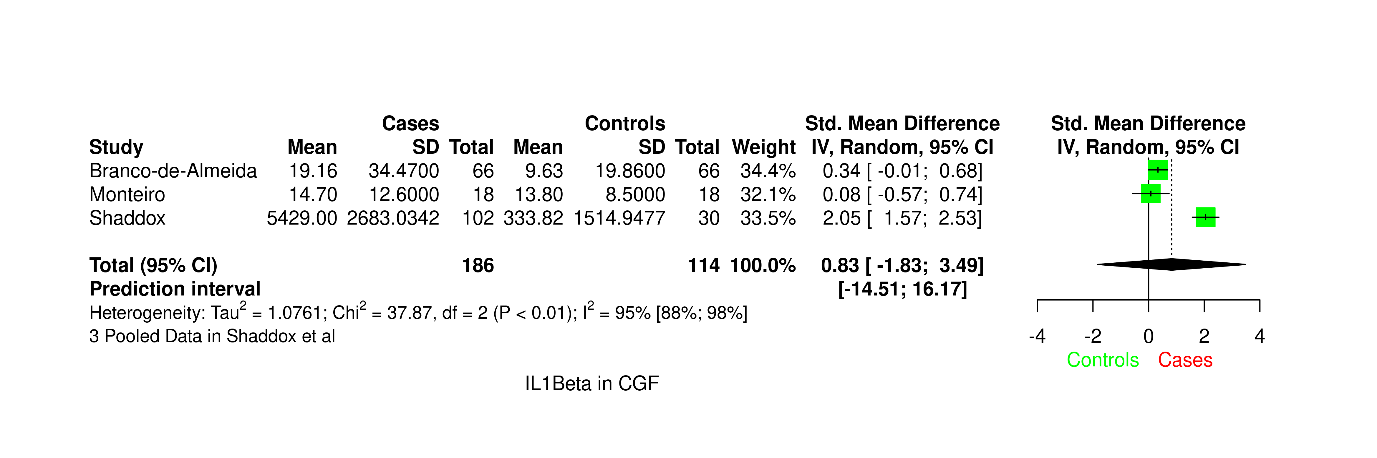

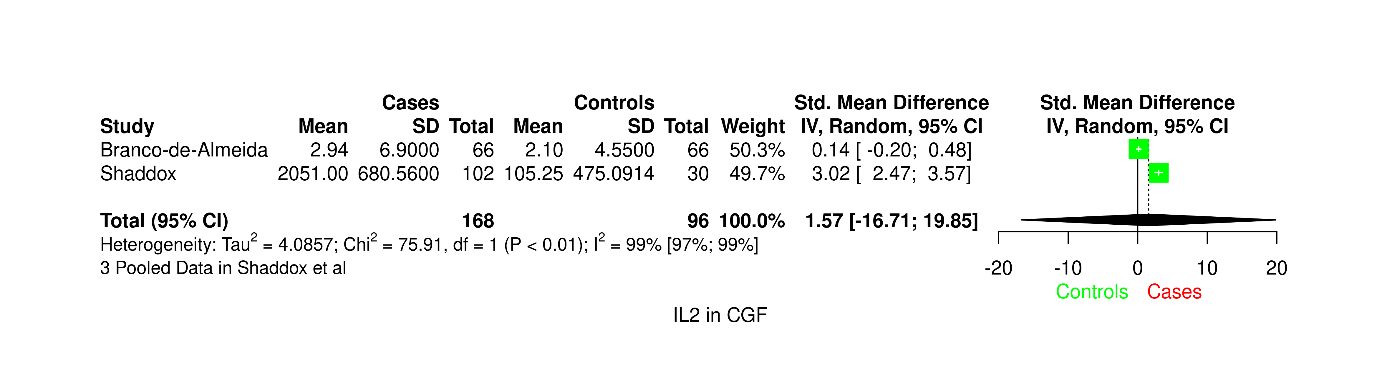

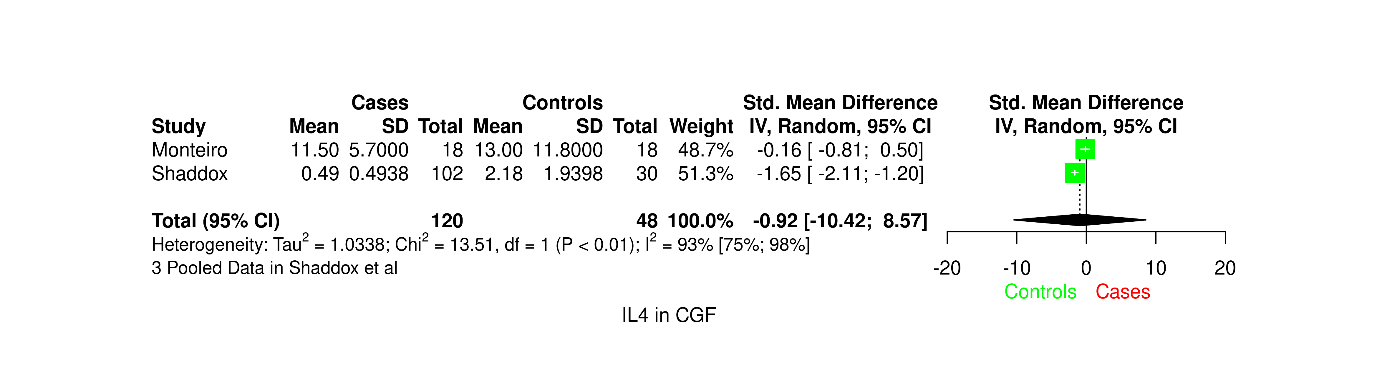

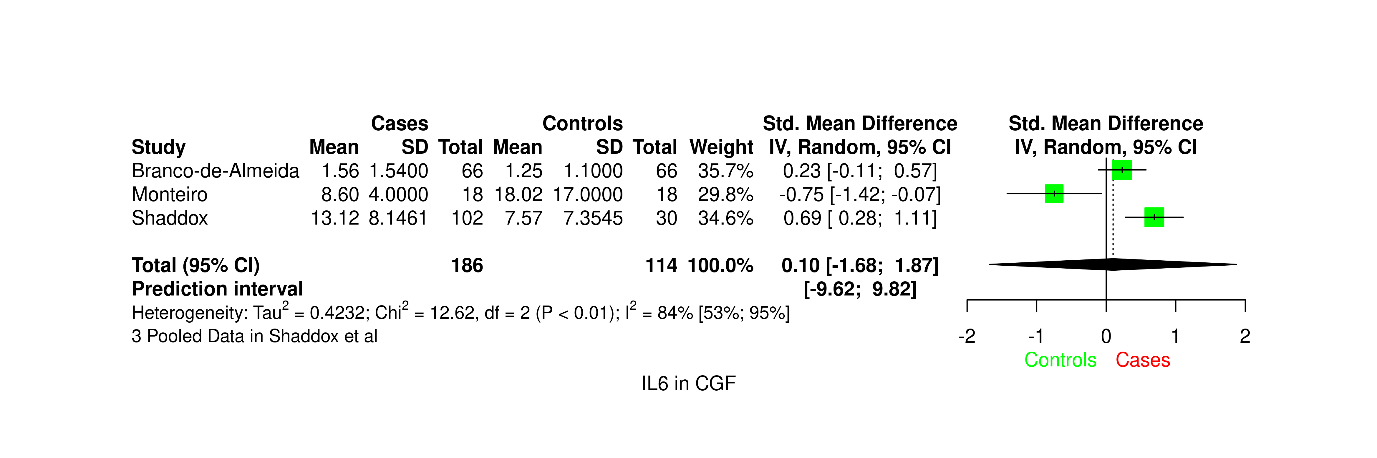

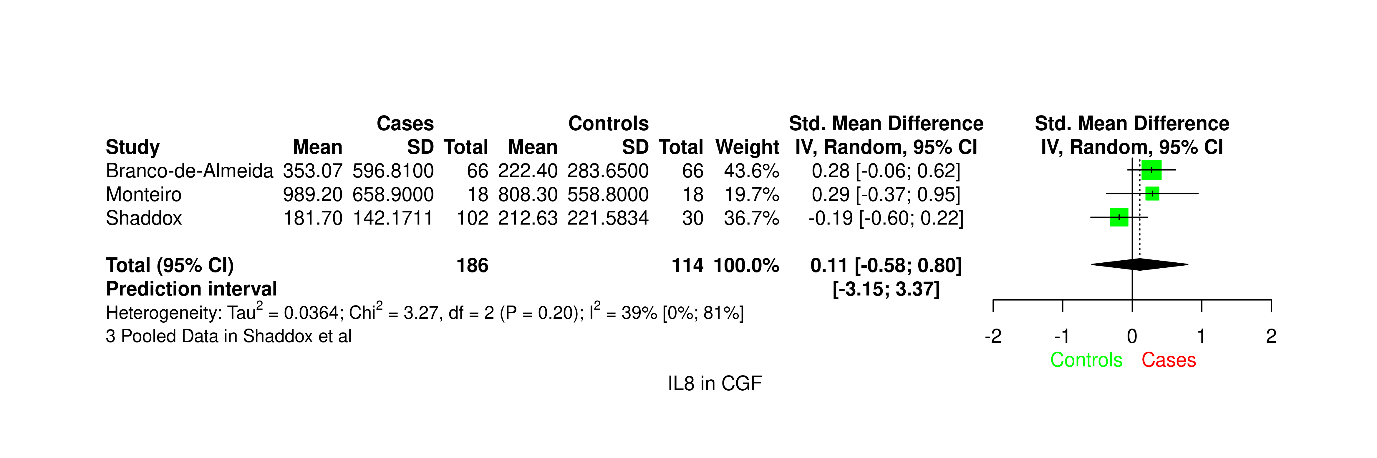

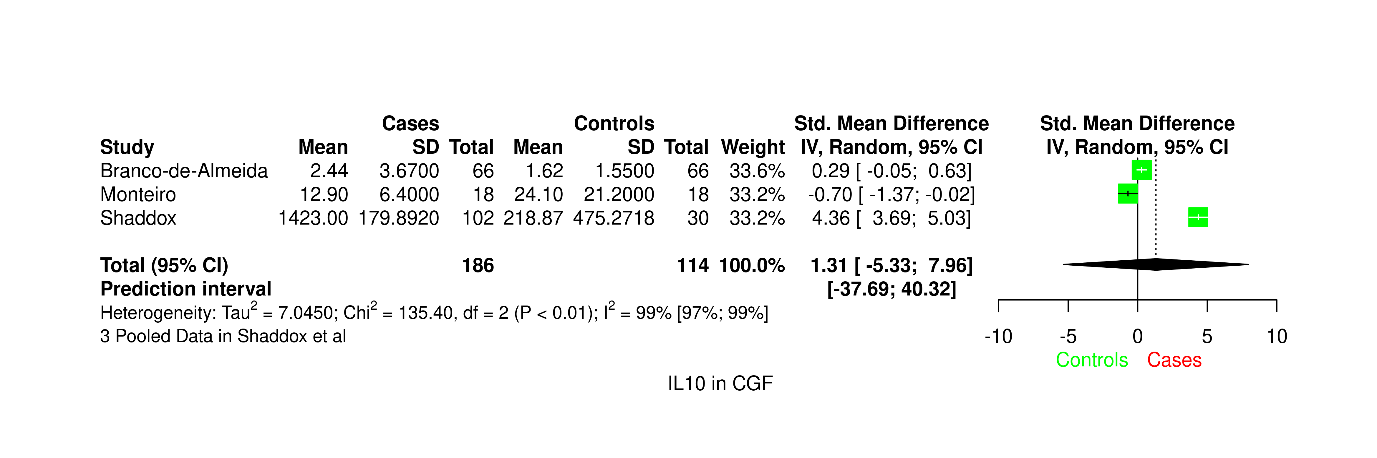

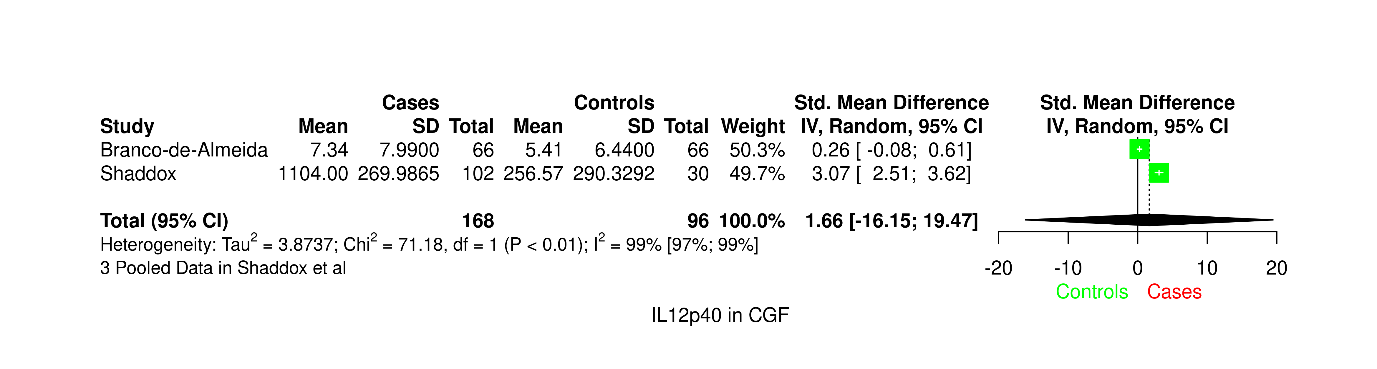

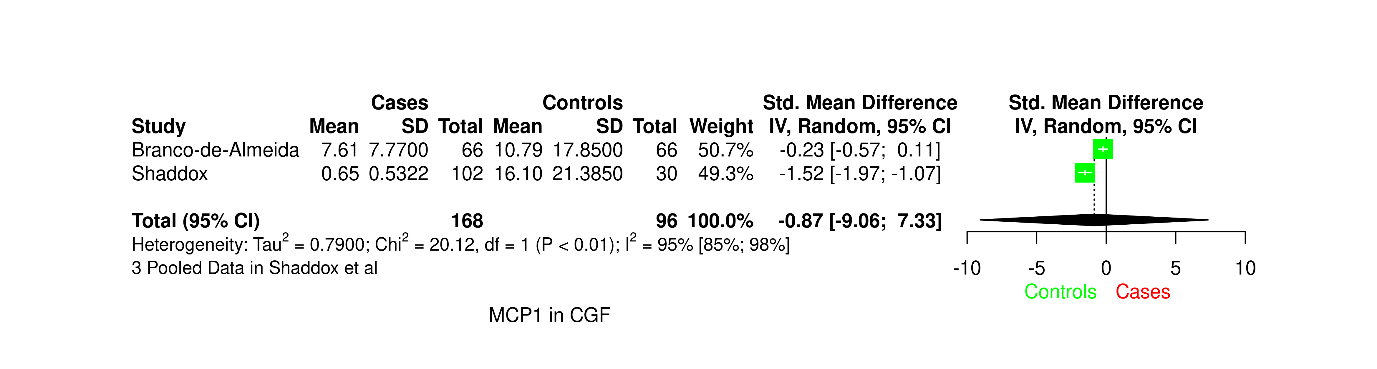

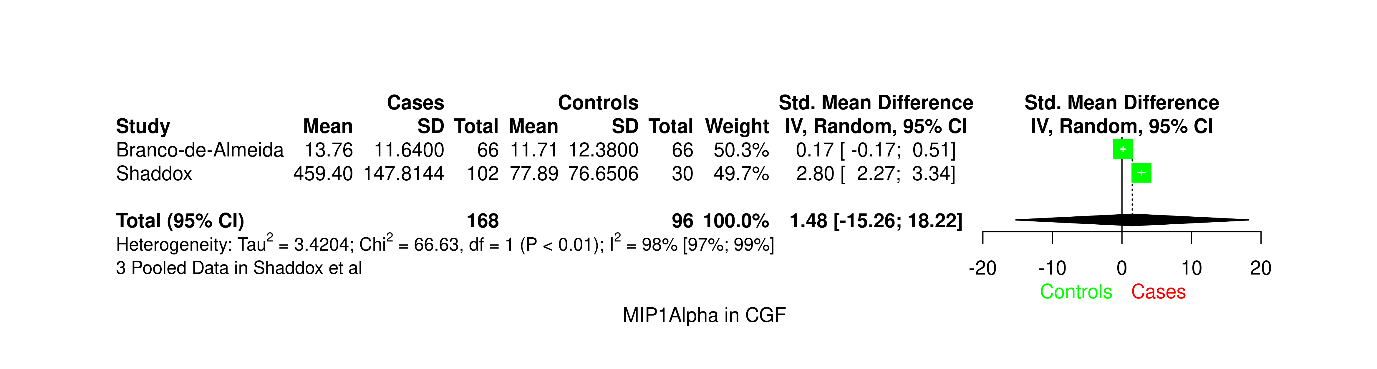

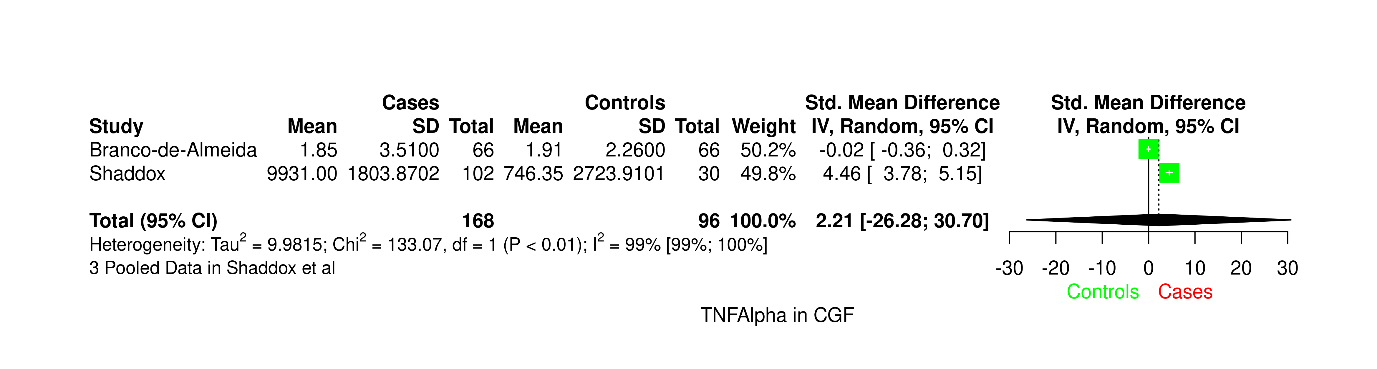

Supplement: Supplementary file 4 — Supplementary file4 (DOCX 1214 KB) [file 784_2023_5169_MOESM4_ESM.docx]

**Appendix 4. Six meta-analysis models were performed for biomarkers in serum samples.**

**
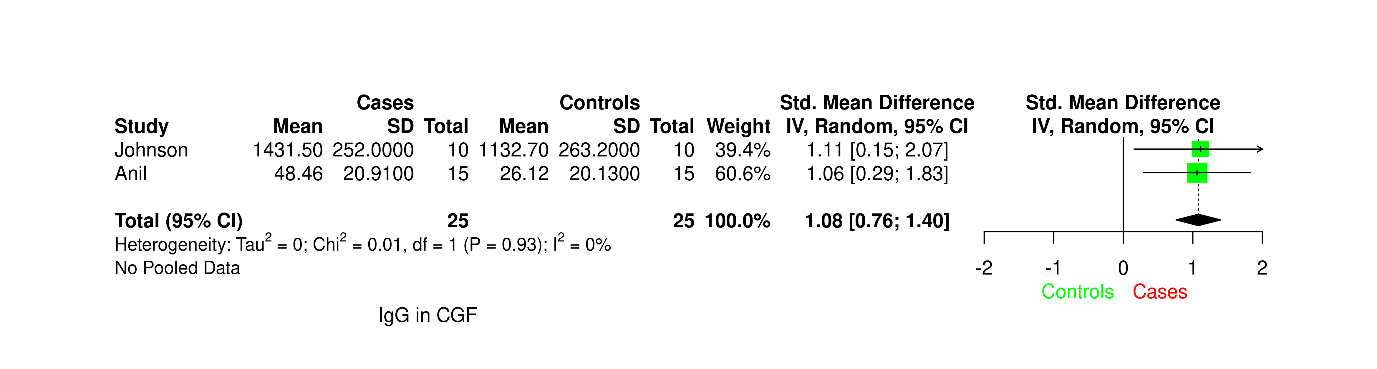

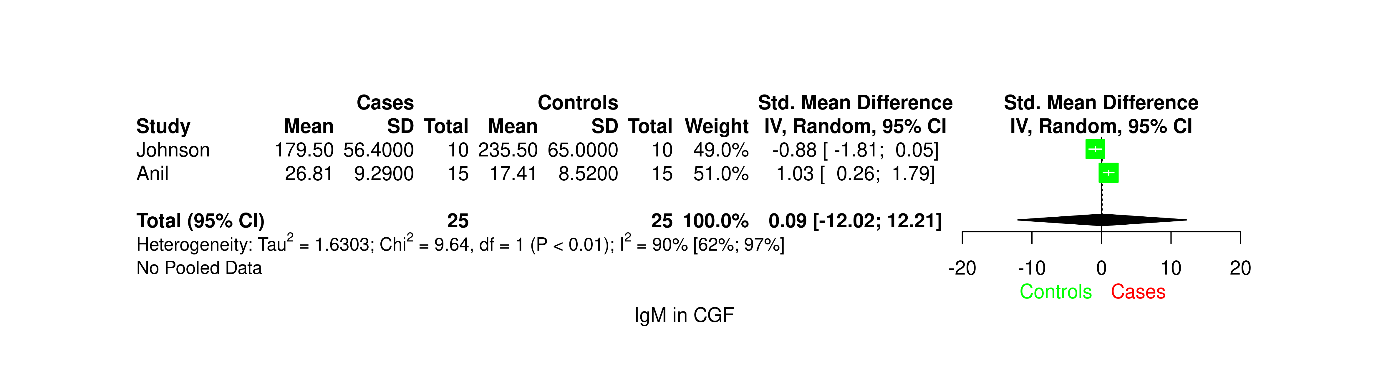
**


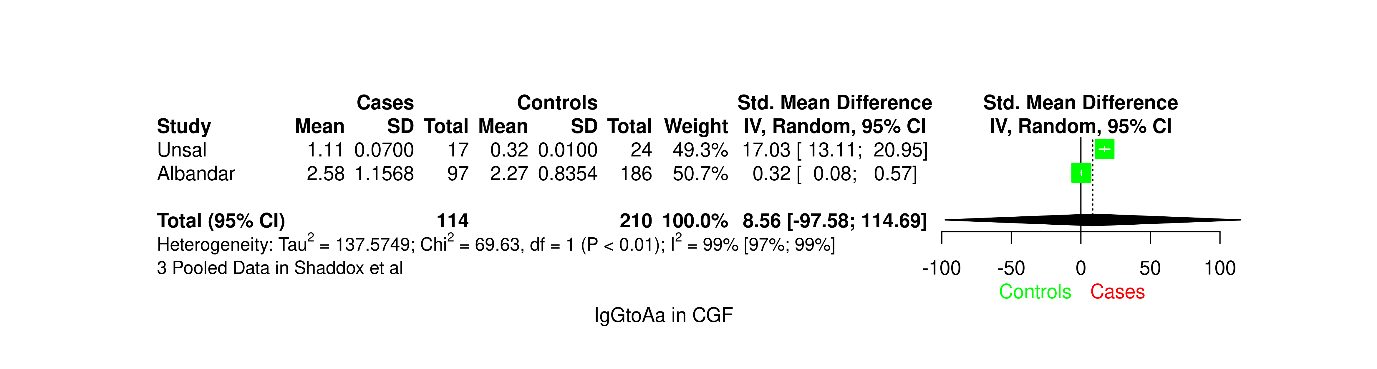

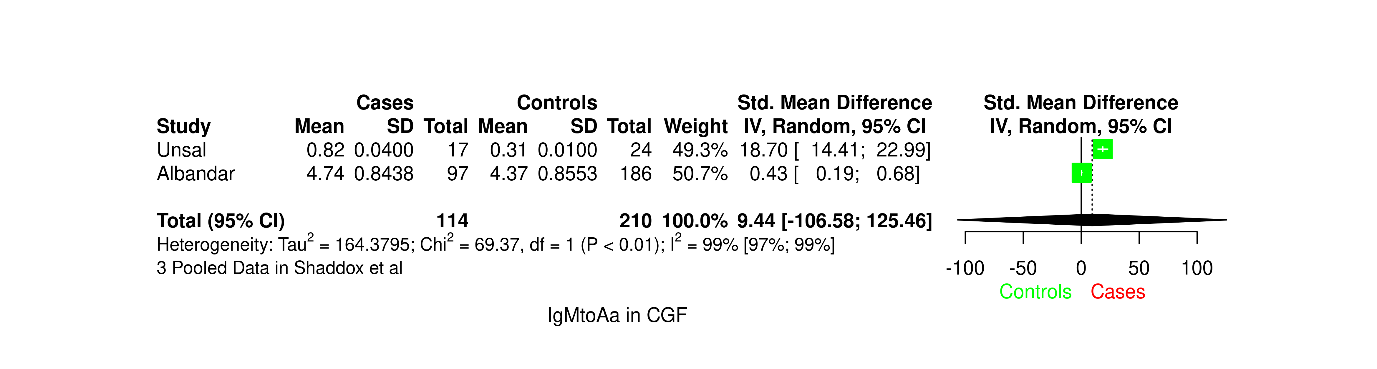

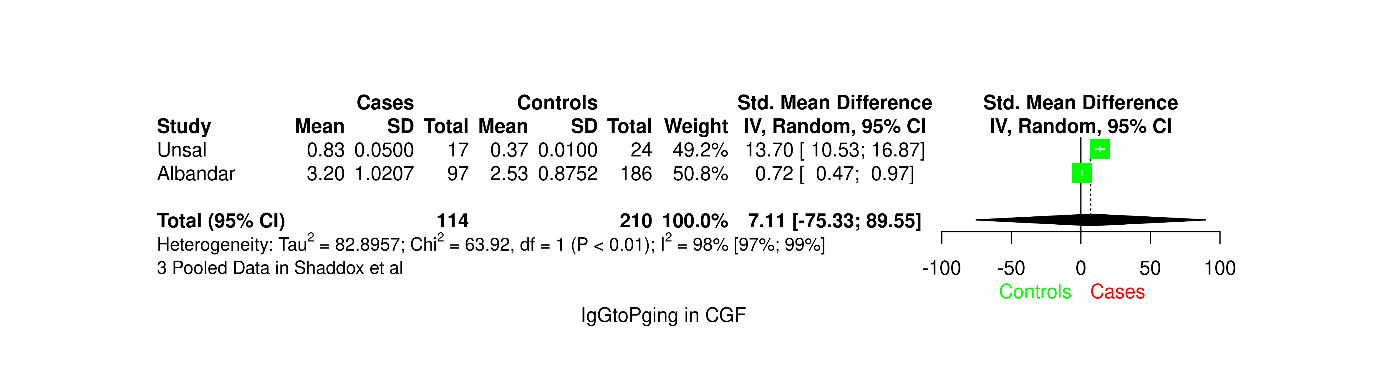


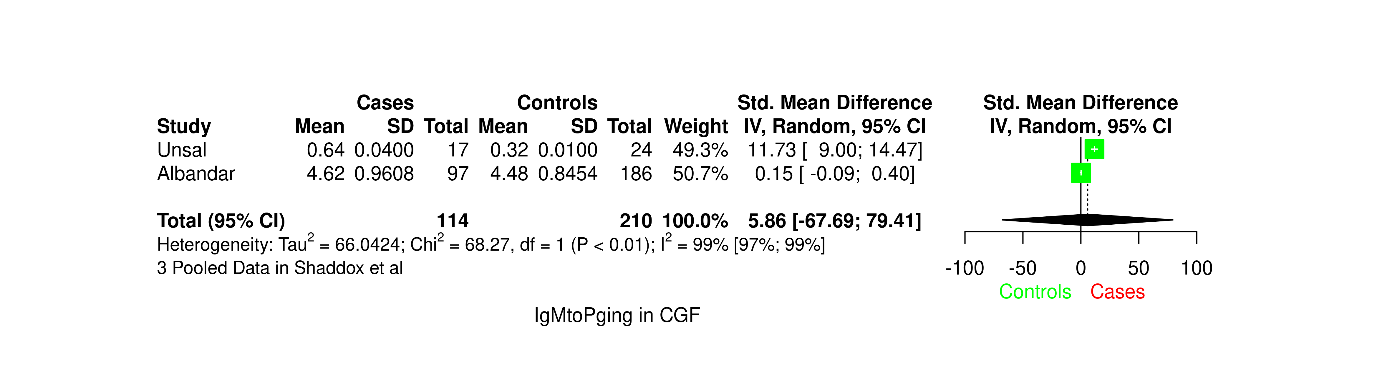

Supplement: Supplementary file 5 — Supplementary file5 (DOCX 553 KB) [file 784_2023_5169_MOESM5_ESM.docx]
