# Supplementary material for: Biomarkers for diagnosis of stage III, grade C with molar incisor pattern periodontitis in children and young adults: a systematic review and meta-analysis
Source: Clin Oral Investig. 2023 Aug 3;27(9):4929–55. doi: 10.1007/s00784-023-05169-x (PMC10492694; doi:10.1007/s00784-023-05169-x)
Supplement: Supplementary file 6 — Supplementary file6 (DOCX 39 KB) [file 784_2023_5169_MOESM6_ESM.docx]

**Appendix 6. Risk of bias of the 28 studies**

| **Cohort studies** | **S1** | **S2** | **S3** | **S4** | **C1** | **O1** | **O2** | **O3** | **Total** | **Quality** |
| --- | --- | --- | --- | --- | --- | --- | --- | --- | --- | --- |
| Branco-de-Almeida, 2020 | 1 | 1 | 1 | 1 | 2 | 1 | 1 | 1 | 9 | Good |
| Albandar,1998 | 1 | 1 | 1 | 1 | 2 | 1 | 1 | 1 | 9 | Good |
| Goncxalves,2013 | 1 | 1 | 1 | 1 | 2 | 1 | 1 | 1 | 9 | Good |
| Kalash,2015 | 1 | 1 | 1 | 1 | 2 | 1 | 1 | 1 | 9 | Good |
| **Case-Control studies** | **S1** | **S2** | **S3** | **S4** | **C1** | **E1** | **E2** | **E3** | **Total** | **Quality** |
| Akalin,1993 | 0 | 1 | 1 | 1 | 0 | 1 | 1 | 1 | 6 | Good |
| Celenligil, 1990 | 1 | 1 | 1 | 1 | 0 | 1 | 1 | 1 | 7 | Good |
| Dibart, 1998 | 1 | 1 | 1 | 1 | 0 | 1 | 1 | 1 | 7 | Good |
| Monteiro,2020 | 1 | 1 | 1 | 1 | 2 | 1 | 1 | 1 | 9 | Good |
| Schenck,1989 | 1 | 1 | 1 | 1 | 1 | 1 | 1 | 1 | 8 | Good |
| Shaddox,2011 | 1 | 1 | 1 | 1 | 2 | 1 | 1 | 1 | 9 | Good |
| Acquier,2017 | 1 | 1 | 1 | 0 | 2 | 1 | 1 | 1 | 8 | Good |
| Acquier,2015 | 1 | 1 | 1 | 0 | 2 | 1 | 1 | 1 | 8 | Good |
| Johnson,1980 | 1 | 1 | 1 | 1 | 0 | 1 | 1 | 1 | 7 | Good |
| Albandar,2001 | 1 | 1 | 1 | 1 | 1 | 1 | 1 | 1 | 8 | Good |
| Albandar,2002 | 1 | 1 | 1 | 1 | 2 | 1 | 1 | 1 | 9 | Good |
| Alfant,2008 | 1 | 1 | 1 | 1 | 1 | 1 | 1 | 1 | 8 | Good |
| Anil,1990 | 1 | 1 | 1 | 1 | 2 | 1 | 1 | 1 | 9 | Good |
| Celenligir,1998 | 1 | 1 | 1 | 1 | 0 | 1 | 1 | 1 | 7 | Good |
| Fine,2013 | 0 | 1 | 1 | 0 | 1 | 1 | 1 | 1 | 6 | Fair |
| Friedman,1983 | 1 | 1 | 1 | 1 | 0 | 1 | 1 | 1 | 7 | Good |
| Lehner,1974 | 1 | 1 | 1 | 0 | 1 | 1 | 1 | 1 | 7 | Good |
| Sjödin,1995 | 1 | 1 | 1 | 1 | 1 | 1 | 1 | 1 | 8 | Good |
| Unsal,1996 | 1 | 1 | 1 | 1 | 0 | 1 | 1 | 1 | 7 | Good |
| Zafiropoulos,1987 | 0 | 1 | 1 | 1 | 2 | 1 | 1 | 1 | 8 | Good |
| **Cross-sectional** | **S1** | **S2** | **S3** |  | **C1** | **O1** | **O2** |  | **Total** | **Quality** |
| Bartova,1995 | 1 | 0 | 0 |  | 0 | 1 | 1 |  | 3 | High Risk |
| Sandholm,1983 | 1 | 0 | 0 |  | 0 | 1 | 1 |  | 3 | High Risk |
| Spindler,1985 | 1 | 0 | 0 |  | 0 | 1 | 1 |  | 3 | High Risk |
| Tavakoli, 2022 | 1 | 0 | 0 |  | 2 | 1 | 1 |  | 5 | High risk |

**NEWCASTLE - OTTAWA QUALITY ASSESSMENT SCALE**

**COHORT STUDIES**

Note: A study can be awarded a maximum of one star for each numbered item within the Selection and Outcome categories. A maximum of two stars can be given for Comparability

**Selection**

1) Representativeness of the exposed cohort

a) truly representative of the average **AgP Pt.** (describe) in the community **🟑**

b) somewhat representative of the average ______________ in the community **🟑**

c) selected group of users eg nurses, volunteers

d) no description of the derivation of the cohort

2) Selection of the non-exposed cohort

**a)** drawn from the same community as the exposed cohort **🟑**

b) drawn from a different source

c) no description of the derivation of the non-exposed cohort

3) Ascertainment of exposure

**a)** secure record (eg. surgical records) **🟑**

b) structured interview **🟑**

c) written self-report

d) no description

4) Demonstration that outcome of interest was not present at start of study (Tx. Of disease)

a) yes **🟑**

b) no

**Comparability**

1) Comparability of cohorts on the basis of the design or analysis

a) study controls for **age** (select the most important factor) **🟑**

b) study controls for any additional factor **🟑Gender** (This criteria could be modified to indicate specific control for a second important factor.)

**Outcome**

1) Assessment of outcome

a) independent blind assessment **🟑**

b) record linkage **🟑**

c) self report

d) no description

2) Was follow-up long enough for outcomes to occur (6 months=a, if not then b)

a) yes (select an adequate follow up period for outcome of interest) **🟑**

b) no

3) Adequacy of follow up of cohorts

a) complete follow up - all subjects accounted for **🟑**

b) subjects lost to follow up unlikely to introduce bias - small number lost - > **10 %** (select an adequate %) follow up, or description provided of those lost) **🟑**

c) follow up rate **< 10%** (select an adequate %) and no description of those lost

d) no statement

| Cohort | Good quality | Fair | Poor |
| --- | --- | --- | --- |
| Selection | 3 or 4 | 2 | 0 or 1 |
| Comparability | 1 or 2 | 1 or 2 | 0 |
| Outcome | 2 or 3 | 2 or 3 | 0 or 1 |

**NEWCASTLE - OTTAWA QUALITY ASSESSMENT SCALE**

**CASE CONTROL STUDIES**

Note: A study can be awarded a maximum of one star for each numbered item within the Selection and Exposure categories. A maximum of two stars can be given for Comparability.

**Selection**

1) Is the case definition adequate?

a) yes, with independent validation **🟑**

b) yes, eg record linkage or based on self-reports

c) no description

2) Representativeness of the cases

a) consecutive or obviously representative series of cases **🟑**

b) potential for selection biases or not stated

3) Selection of Controls

a) community controls **🟑**

b) hospital controls

**c)** no description

4) Definition of Controls

a) no history of disease (endpoint) **🟑**

b) no description of source

**Comparability**

1) Comparability of cases and controls on the basis of the design or analysis

a) study controls for **age** (Select the most important factor.) **🟑**

b) study controls for any additional factor **🟑 Gender** (This criteria could be modified to indicate specific control for a second important factor.)

**Exposure**

1) Ascertainment of exposure

a) secure record (eg surgical records) **🟑**

b) structured interview where blind to case/control status **🟑**

c) interview not blinded to case/control status

d) written self report or medical record only

e) no description

2) Same method of ascertainment for cases and controls

a) yes **🟑**

b) no

3) Non-Response rate (dropout)

a) same rate for both groups **🟑**

b) non respondents described

c) rate different and no designation

| Case-Control | Good quality | Fair | Poor |
| --- | --- | --- | --- |
| Selection | 3 or 4 | 2 | 0 or 1 |
| Comparability | 1 or 2 | 1 or 2 | 0 |
| Outcome | 2 or 3 | 2 or 3 | 0 or 1 |

**NEWCASTLE - OTTAWA QUALITY ASSESSMENT SCALE**

**CROSS-SECTIONALSTUDIES**

**Selection (maximum 3 points)**

1) Representativeness of the sample: 1 point was given if the sample was truly representative of the average in the target population (all subjects or random sampling) or somewhat representative (non-random sampling)

2) Sample size: 1 point was given if sample size was justified and satisfactory

3) Non-included subjects: 1 point was given if comparability between included and non-included subjects was established, and if the inclusion rate was satisfactory.

**Comparability (maximum 2 points)**

Subjects in different outcome groups are comparable, based on study design or analysis. Confounding factors are controlled.

1) One point was given if there was adequate adjustment of anthropometric measures for age=1 and gender, and adequate adjustment of endocrine disrupting chemical urinary levels for urinary creatinine levels (or of endocrine disrupting serum levels for serum lipid levels).

2) One additional point was given if there was adequate adjustment for other factors influencing measures of body weight or adiposity, such as race/ethnicity, poverty-to income ratio, caloric intake, educational level and exercise habits.

**Outcome (maximum 3 points)**

**1)** Assessment of the outcome: 1 point were given if the measures of body weight (body mass index, waist circumference) or body fat were assessed independently and blindly or from record linkage (database records), and 1 point was given if the outcome was assessed by self-report

**2)** Statistical test: 1 point was given if the statistical test used to analyze the data was clearly described and appropriate, and the measurement of the association was presented, including confidence intervals and the probability level (p value).

| Cross-sectional | Low risk | Medium risk | High risk |
| --- | --- | --- | --- |
| Total | 8 or 7 | 6 | 5 or less |
| Selection | 3 | 1-2 | 0 |
| Comparability | 2 | 1 | 0 |
| Outcome | 3 | 2 | 1 |

Ribeiro CM, et al. BMJ Open 2020; 10:e033509. doi: 10.1136/bmjopen-2019-033509
